# Characterization of young and aged ferrets as animal models for SARS-CoV-2 infection with focus on neutrophil extracellular traps

**DOI:** 10.3389/fimmu.2023.1283595

**Published:** 2023-12-15

**Authors:** Veronika Pilchová, Ingo Gerhauser, Federico Armando, Katrin Wirz, Tom Schreiner, Nicole de Buhr, Gülşah Gabriel, Kerstin Wernike, Donata Hoffmann, Martin Beer, Wolfgang Baumgärtner, Maren von Köckritz-Blickwede, Claudia Schulz

**Affiliations:** ^1^Research Center for Emerging Infections and Zoonoses (RIZ), University of Veterinary Medicine Hannover, Foundation, Hannover, Germany; ^2^Institute of Biochemistry, University of Veterinary Medicine Hannover, Foundation, Hannover, Germany; ^3^Department of Pathology, University of Veterinary Medicine Hannover, Foundation, Hannover, Germany; ^4^Center for Systems Neuroscience Hannover (ZSN), University of Veterinary Medicine Hannover, Foundation, Hannover, Germany; ^5^Department for Viral Zoonoses-One Health, Leibniz Institute of Virology, Hamburg, Germany; ^6^Institute for Virology, University for Veterinary Medicine Hannover, Foundation, Hannover, Germany; ^7^Institute of Diagnostic Virology, Friedrich Loeffler Institute, Greifswald, Germany

**Keywords:** age, animal model, COVID-19, ferret, NETs, SARS-CoV-2, virus

## Abstract

Neutrophil extracellular traps (NETs) are net-like structures released by activated neutrophils upon infection [e.g., severe acute respiratory syndrome coronavirus 2 (SARS-CoV-2)] as part of the innate immune response that have protective effects by pathogen entrapment and immobilization or result in detrimental consequences for the host due to the massive release of NETs and their impaired degradation by nucleases like DNase-1. Higher amounts of NETs are associated with coronavirus disease 2019 (COVID-19) severity and are a risk factor for severe disease outcome. The objective of our study was to investigate NET formation in young versus aged ferrets to evaluate their value as translational model for SARS-CoV-2-infection and to correlate different NET markers and virological parameters. In each of the two groups (young and aged), nine female ferrets were intratracheally infected with 1 mL of 10^6^ TCID_50_/mL SARS-CoV-2 (BavPat1/2020) and euthanized at 4, 7, or 21 days post-infection. Three animals per group served as negative controls. Significantly more infectious virus and viral RNA was found in the upper respiratory tract of aged ferrets. Interestingly, cell-free DNA and DNase-1 activity was generally higher in bronchoalveolar lavage fluid (BALF) but significantly lower in serum of aged compared to young ferrets. In accordance with these data, immunofluorescence microscopy revealed significantly more NETs in lungs of aged compared to young infected ferrets. The association of SARS-CoV-2-antigen in the respiratory mucosa and NET markers in the nasal conchae, but the absence of virus antigen in the lungs, confirms the nasal epithelium as the major location for virus replication as described for young ferrets. Furthermore, a strong positive correlation was found between virus shedding and cell-free DNA or the level of DNAse-1 activity in aged ferrets. Despite the increased NET formation in infected lungs of aged ferrets, the animals did not show a strong NET phenotype and correlation among tested NET markers. Therefore, ferrets are of limited use to study SARS-CoV-2 pathogenesis associated with NET formation. Nevertheless, the mild to moderate clinical signs, virus shedding pattern, and the lung pathology of aged ferrets confirm those animals as a relevant model to study age-dependent COVID-19 pathogenesis.

## 1 Introduction

Severe acute respiratory syndrome coronavirus 2 (SARS-CoV-2), the etiologic agent of coronavirus disease 2019 (COVID-19), caused a global pandemic leading to health system crisis and 7 million deaths worldwide (1). A mild course of an infection typically includes cough, fever, malaise, and diarrhea, while during a moderate illness, additional signs of lower respiratory disease occur. Severe cases often suffer from acute respiratory distress syndrome (ARDS), multiple organ failure, coagulopathy, and impaired adaptive immunity (2). Older age is, among other associated factors like sex or other comorbidities, the most prominent risk factor leading to severe COVID-19 outcomes and increased morbidity and mortality (3). Young children usually stay asymptomatic or show only mild symptoms, while elderly, especially those above 65 years of age, show an increased case fatality rate (4).

Animal models are crucial for the understanding of pathogenesis of the SARS-CoV-2 infection, development of therapeutics, and vaccine testing. Ferrets (*Mustela putorius furo*) are considered to be the most suitable animal model for influenza virus research due to their physiological and immunological similarities with humans (5). Therefore, ferrets were also tested as an animal model for SARS-CoV-2 infection, showing their natural susceptibility (6–8) and an ability for robust SARS-CoV-2 transmission by air (9–13). Additionally, ferrets were confirmed to be a relevant model for SARS-CoV-2 adaptive immunity (14, 15), pathogenesis (16, 17), vaccine (1), and drug treatment research (18, 19).

Based on clinical data from human patients and based on data derived from hamster experiments, it is well known that the innate immune response to a severe course of SARS-CoV-2 infection includes a massive infiltration of neutrophils into the respiratory tissues. Activated neutrophils can then form neutrophil extracellular traps (NETs), which may contribute to detrimental effects of infiltrating neutrophils (20–24). NETs are released by activated neutrophils into the extracellular space and consist of DNA fibers with associated proteins, e.g., histones, myeloperoxidase, and cationic antimicrobial peptides (AMPs) such as cathelicidins. Thus, for the detection of NETs, DNA/histone-1 complexes, myeloperoxidase (MPO), and elevated levels of cell-free DNA are used in combination as typical biomarkers (25). NETs were originally described as a host’s protective mechanism against infection by pathogen entrapment (26). For clearance of NETs, DNA fibers are degraded by host nucleases, since their accumulation can lead to detrimental effects (27). In fact, overwhelming NET formation during SARS-CoV-2 infection and released NET components can lead to immunothrombosis, tissue damage, vascular leakage, and acute respiratory distress syndrome (ARDS) (24, 28–31). Furthermore, increased levels of NET markers circulating in serum of COVID-19 patients with moderate or severe disease compared to sera from healthy individuals were described (32). Therefore, NETs are recently discussed as target for therapeutic interventions (24, 28, 31). The aim of this study was to characterize young and aged ferrets as potential animal models for SARS-CoV-2 infection with focus on NET formation.

## 2 Materials and methods

### 2.1 Virus and cell culture

SARS-CoV-2 human isolate (Human 2019-nCov ex China, BavPat1/2020, Ref-SKU 026V-03883, kindly provided by Christian Drosten, Charité, Berlin, Germany) was passaged once on Vero E6 cells at 37°C and 5% CO_2_ using Dulbecco’s modified Eagle’s medium (DMEM; Gibco, Thermo Fisher Scientific Inc., USA) with 1% penicillin/streptomycin (Pen/Strep, Sigma), 1% GlutaMAX-I (GibcoTM, Thermo Fisher Scientific Inc.), and 2% of heat-inactivated fetal bovine serum (FBS; SUPERIOR stabil, Bio&SELL GmbH). All experiments were conducted at room temperature under biosafety level (BSL)-3 conditions in laboratories with a negative pressure of −75 Pa.

### 2.2 Animals

Young (8 months) and aged (3–3.5 years) female ferrets were purchased from a commercial breeder and randomly allocated into open cages (Ferret suite, Tecniplast) in groups of three according to the animal identification number and day of euthanasia. An overview of animal numbers and groups are shown in **Supplementary Table S1**. All animals were housed in the Research Center for Emerging Infections and Zoonoses under BSL-3 conditions 1 month before the animal experiment with a 12-h light and dark cycle regime. Ferrets received water and food *ad libitum*. Before the experiment, all animals tested negative by virological and serological analyses (Friedrich-Loeffler-Institut, Greifswald - Insel Riems, Germany), excluding a previous SARS-CoV-2 infection. Health monitoring of the ferrets showed that the animals were free of a previous infection testing a wide range of selected pathogens (**Supplementary Table S2**). Analyses of (respiratory) bacterial and viral pathogens included *Carnivore amdoparvovirus 1* (Aleutian disease), SARS-CoV-2, *Bordetella bronchiseptica, Streptococcus pneumoniae*, *Staphylococcus areus*, *Pasteurella multocida* genome, and SARS-CoV-2 and influenza virus antibodies. Ferret enteric coronavirus, Cryptosporidia, and *Eimeria furonis* were detected in pooled fecal samples of young ferrets, while results of aged ferrets revealed that they were specific pathogen free for all tested pathogens (**Supplementary Table S2**). The breeder stated regular testing for *Helicobater mustelae* to be negative. Aged animals received a Suprelorin 4.7 mg implant (Virbac) 2 weeks before the experiment.

### 2.3 Study design

Young children and adolescents usually stay asymptomatic or show only mild symptoms (33), while elderly (humans), especially those above 65 years of age, show an increased severity in clinical signs and case fatality rate after SARS-CoV-2 infection (3, 4). Accordingly, the respective age groups in ferrets were determined based on several factors: i) the sexual maturity of ferrets starts at age of 6–12 months, ii) the breeding period is 2–5 years, and iii) the life expectancy is 5–11 years, according to (34, 35). We chose “young” ferrets around the age of sexual maturity (8 months old). If assuming a life expectancy of 84 years in women (data from Western Europe in 2023: https://www.worlddata.info/life-expectancy.php), taking 5 years of age as threshold for “old” ferrets, and calculating a relative age of ferrets compared to humans with an age of 65 years, the age of ferrets would be 3.87 years of age (=[5/84] × 65). Therefore, similarly as Kim et al. (36), we chose 3–3.5-years-old ferrets for the “aged” group. We define “old” ferrets as ferrets of an age of 5 years or older due to their life expectancy of 5–11 years.

Furthermore, we chose females for our study, since according to the EU regulation 2010/63/EU CETS Annex III (Requirements for establishments and for the care and accommodation of animals) (37), females require considerably less space in cages then males. This allows more animals in each trial due to limitations in space in an animal stable. Females are generally smaller and lighter and are therefore easier to handle. Intact males may get aggressive during the breading period due to an increased sex drive and cannot be kept together during that time (34, 35). For example, in the study of van de Ven (14), it was not possible to randomly allocate individual animals to treatment groups due to the strict hierarchy present in male ferret groups.

**Figure 1:**
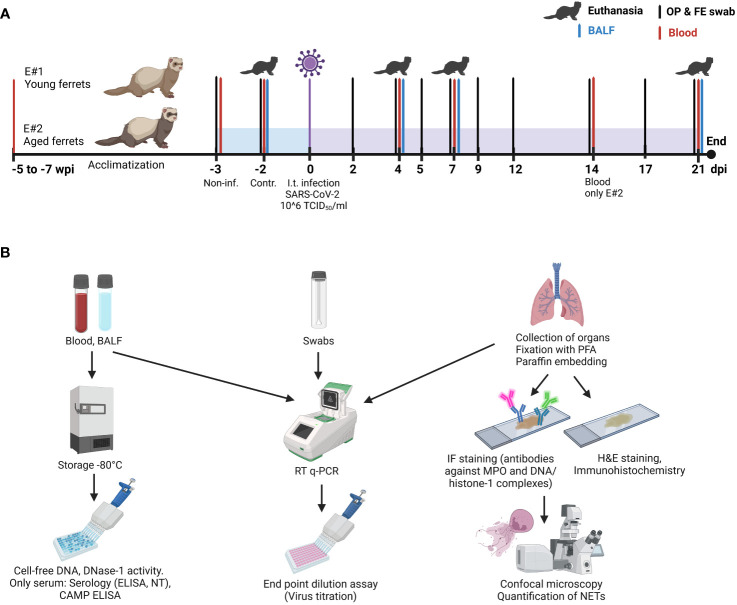
Study design. **(A)** Timeline and sample collection points. “Non-inf.” samples: samples taken from all animals 3 days before infection. “Control” samples: samples taken from three control ferrets euthanatized 2 days before infection. **(B)** Overview of samples and their further downstream analysis. IF, immunofluorescence; E#, experiment number; dpi, days post-infection; wpi, weeks post-infection; H&E, hematoxylin/eosin; NT, neutralization test; PFA, paraformaldehyde; MPO, myeloperoxidase; CAMP, cathelicidin antimicrobial peptide. Created with BioRender.com (Agreement number: RQ25RA8W7D).

This study was conducted in two separate parts, part 1 (E#1) with 12 young (8 months old) ferrets (F1 to F12) and part 2 (E#2) with 12 aged (3–3.5 years old) female ferrets (F13 to F24) (**Figure 1A**, **Supplementary Table S1**).

In each experiment, four groups with each three animals were designed: uninfected control (euthanized 2 days before infection) and SARS-CoV-2-infected groups euthanized at 4, 7, and 21 days post-infection (dpi). Blood samples from ferrets were collected 5 (aged) to 7 (young) weeks and 3 days (young and aged) before infection under anesthesia and additionally from each group on the day of the euthanasia.

Two days before infection, three ferrets in each experiment were euthanized as controls without receiving any vehicle. On day 0, ferrets were infected intratracheally with 1 ml of 10^6^ TCID_50_/mL of SARS-CoV-2. Ferrets were randomly categorized into selected groups. At the experimental endpoints (2 days before infection for controls; 4, 7, or 21 days after infection for SARS-CoV-2 infected ferrets) groups of three ferrets were anesthetized (10 mg/kg ketamine, 0.5 mg/kg midazolam, and 0.1 mg/kg medetomidine) and euthanized by exsanguination (incision in the aorta and caudal vena cava).

### 2.4 Sample collection

Samples were collected as described before (38) and as shown in **Figure 1A** (study design). Briefly, oropharyngeal and fecal swabs were taken 3 days before SARS-CoV-2 infection and at 2, 4, 5, 7, 9, 12, 14, 17, and 21 dpi. In addition, blood samples were collected before infection (F1–F12) and from a group of three animals on their day of euthanasia: at 4 dpi (F4–F6), 7 dpi (F7–F9), and 21 dpi (F10–F12). Blood samples were also collected at 14 dpi from the aged ferrets F22–F24 in E#2.

Bronchoalveolar lavage fluid (BALF) was obtained in sternal recumbence immediately after euthanasia. An 11-cm catheter was inserted into the trachea using a laryngoscope; 3 mL phosphate-buffered saline (PBS) with 0.2% bovine serum albumin (BSA) and 1% penicillin/streptomycin was injected using a 5-mL syringe, and approximately 1.5 mL was recovered by aspiration. Then, a full necropsy was performed, and tissue samples were taken for further analysis. The overview of the following downstream analysis of all samples is presented in **Figure 1B**.

### 2.5 End-point dilution assay

For virus quantification with end-point dilution assay according to Pilchová et al. (39), Vero E6 cells were seeded into 96-well tissue culture plates (Sarstedt) with DMEM supplemented with 2% FBS, 1% Pen/Strep, and 1% GlutaMAX-I 1 day before the experiment. Samples were 10-fold serially diluted (20 µL virus in 180 µL medium), and cell morphology was checked for cytopathic effect (CPE) at multiple days until 5–7 days post-infection (dpi). Virus titers are given as TCID_50_ (50% tissue culture infective dose) per mL (TCID_50_/mL) or per gram (TCID_50_/g) and were calculated according to the Spearman and Kärber method (40). At least two technical replicates were made from each biological replicate. The results of the end-point dilution assay were calculated according to Binder (41) and as described previously (39). Therefore, the limit of the detection of the assay for respiratory tissue homogenates and for liquid samples (BALF, swabs) was 1,693 TCID_50_/g and 506 TCID_50_/mL, respectively. The detection limit (1,693 TCID_50_/g) of the end-point dilution was calculated using the titration result of the sample multiplied with the tissue supernatant volume of 0.5 mL and divided with the max. weight of all respiratory tissue samples (0.1494 g): [(TCID_50_/mL × 0.5)/weight]. For statistical analysis, the negative samples were calculated as half of the detection limit (847 TCID_50_/g for respiratory tissue homogenates and 253 TCID_50_/mL for liquid samples). In general, all samples positive for viral RNA were tested with end-point dilution assay. Additionally, some PCR-negative samples were selected and tested as tissue culture control.

### 2.6 Quantitative real-time reverse transcription PCR

**Figure 2:**
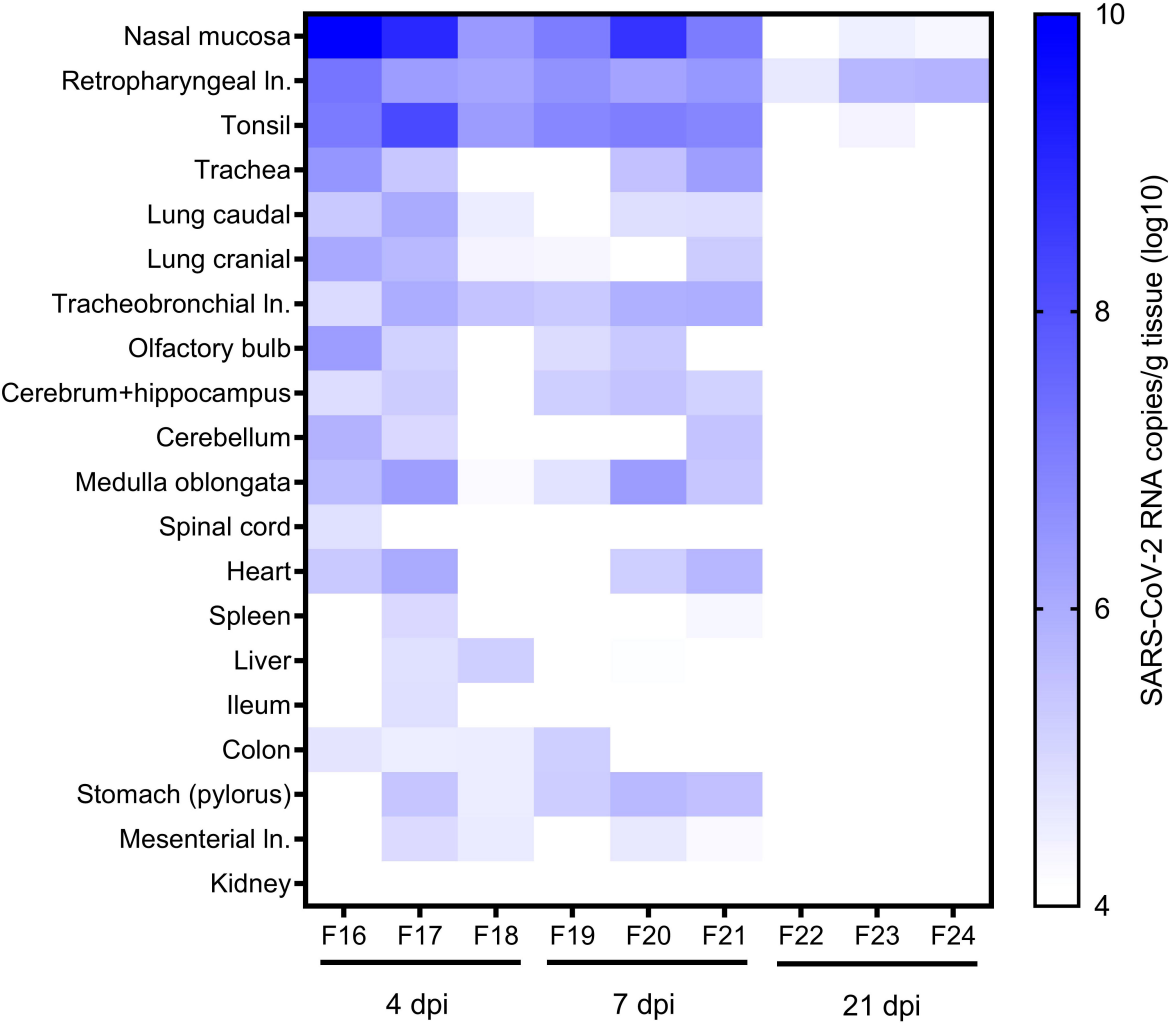
RNA in other organs. SARS-CoV-2 RNA copies/g in various tissue samples of ferrets at 4, 7, and 21 days post-infection (dpi) were quantified with SARS-CoV-2 specific real-time quantitative reverse transcription-PCR detecting the RNA-dependent RNA polymerase (RdRp) (38). ln., lymph node.

Nucleic acid extraction from 100 μL of swab, ethylenediaminetetraacetic acid (EDTA) whole blood, BALF, and tissue homogenate supernatants (elution in 100 µL), and real-time reverse transcription polymerase chain reaction (RT-qPCR) were conducted as described before (38). Briefly, for nucleic acid extraction, the Nucleo MagVet Kit (Macherey-Nagel) with a KingFisher 96 platform (Thermo Fisher Scientific) was used. For RT-qPCR, 2.5 µL of template was amplified using the AgPath-ID™ One-Step RT-PCR Reagents (Thermo Fisher Scientific) with SARS-CoV-2-specific primers and probes targeting the RNA-dependent RNA polymerase (RdRp) gene of SARS-CoV-2 (SARS-2-IP4 assay of Institute Pasteur, recommended by the WHO). An internal control system (EGFP-Mix1) was included. SARS-CoV-2 RNA was amplified and quantified at a CFX96 Touch Real-Time PCR Detection System (Bio- Rad). Quantification cycle (Cq) values ≤ 40 were considered positive (38). As standard, *in vitro* transcribed RNA derived from strain BetaCoV_Wuhan_WIV04_2019 (EPI_ ISL_402124) was used. The transcript contains the amplification regions of the RdRp (“IP2 and IP4”) and E gene as positive strand (kindly provided by Institute Pasteur). The limits of detection of the PCR assays were 2,165 copies/g for all analyzed tissue homogenates (**Figure 2**) and 1,000 copies/mL for the liquid samples (BALF, oropharyngeal, and fecal swabs). As described previously (39), the max. weight (0.2309 g) of the tissue homogenates was used to calculate the detection limits [copies in total sample volume/weight]. For statistical analysis, the negative samples were calculated as half of the detection limit (1,083 copies/g for tissue homogenates and 500 copies/mL for liquid samples).

### 2.7 Serology

SARS-CoV-2-specific antibodies in ferrets were determined before and after intratracheal SARS-CoV-2 infection until 21 dpi with multi-species indirect ELISA (iELISA) and neutralization test (NT) according to (42) (**Supplementary Figure S1**). The iELISA measures SARS-CoV-2-specific antibodies directed against the receptor-binding domain (RBD) of the SARS-CoV-2 S protein (42).

### 2.8 Hematology

EDTA-blood samples were analyzed automatically at a hematology instrument (Vet abc plus, Scilvet) according to the manufacturer’s instructions. The hematology instrument differentiates the cellular components erythrocytes, platelets, and leukocytes including lymphocytes, monocytes, neutrophil, and eosinophil granulocytes. Furthermore, the mean corpuscular volume (MCV), mean corpuscular hemoglobin (MCH), and mean corpuscular hemoglobin concentration (MCHC) of erythrocytes, the hematocrit, and the mean platelet volume (MPV) are determined. Cellular components are measured by impedance and the hemoglobin concentration by photometry.

### 2.9 Histology and immunohistochemistry

Formalin-fixed and paraffin-embedded tissues (2–3-µm sections) were stained with hematoxylin and eosin (HE), and the microscopic examination was conducted by a board-certified veterinary pathologist (IG) as described before (38). For immunohistochemistry, dewaxed and rehydrated sections were incubated in ethanol with 0.5% hydrogen peroxide for 30 min to block endogenous peroxidase. Antigen retrieval was performed by boiling the slides for 20 min in citrate-EDTA buffer (10 mM citrate acid, 2 mM EDTA, 0.05% Tween 20%, pH 6.2) or in citrate buffer (10 mM citrate acid, pH 6.0) using a microwave oven to detect SARS-CoV-2 or ferret systemic coronavirus (FRSCV), respectively. Subsequently, sections were incubated with mouse monoclonal antibodies directed against SARS-CoV-2 nucleocapsid protein (40143-MM05; Sino Biological Europe; 1:3,200 in PBS with 1% BSA and 0.3% TritonX-100) or FRSCV (FIPV3-70; Custom Monoclonals International; 1:10,000 in PBS with 1% BSA) for 1 h at room temperature. Negative control sections were incubated with ascites of Balb/c mice instead of primary antibodies. The lung of a SARS-CoV-2-infected hamster and the liver of a cat with feline infectious peritonitis (FIP) were used as positive tissue controls. Immunolabeling was visualized by the Dako EnVision+ System (K4001; Agilent Technologies) for a goat-anti-mouse secondary antibody (BA-9200; Vector Laboratories; 1:200) and the avidin-biotin-peroxidase complex (ABC) method (PK6100; Vector Laboratories) with 3,3-diaminobenzidine (DAB) as substrate, respectively. Finally, sections were counterstained with Mayer’s hematoxylin. Photos of stained sections were generated by a digital camera mounted on an Olympus BX51 microscope (Olympus Deutschland GmbH) using the CellSens Standard 1.18 software (Olympus Deutschland GmbH).

### 2.10 Scanning electron microscopy

At necropsy, samples from the nasal cavity (cranial and caudal conchae) were collected for scanning electron microscopy (SEM). Samples were fixed in 5% glutaraldehyde buffered with 0.1 M cacodylate buffer (Serva Electrophoresis) and were subsequently embedded by a modified osmium (O)-thiocarbohydrazide (T)-embedding (OTOTO) protocol, followed by critical point drying and coating with gold in a sputter coater (SCD040, Oerlikon Balzers), as described previously. Embedded samples were mounted on 0.5ʺ aluminum specimen stubs (Agar Scientific) using 12 mm Leit-Tabs (Plano) and conductive carbon cement after Göcke (Plano) and examined using a Zeiss EVO 15 scanning electron microscope (Carl Zeiss Microscopy) operating with 10 kV.

### 2.11 Immunofluorescence staining and microscopy of histological sections

For NET detection, paraffin sections from animals at 4 dpi (serial cuts to HE and virus antigen staining) were analyzed. The immunofluorescence staining of paraffin section was performed as described (38). Briefly, mouse IgG2a anti-DNA/histone-1 complexes (MAB3864, Millipore; 0.55 mg; 1:500) in blocking buffer was incubated overnight at 4°C. As secondary antibodies, goat anti-mouse antibody (Alexa488PLUS, Thermo Fisher Scientific) was diluted 1:500 in blocking buffer. Nuclei were stained with Hoechst 33342 (Sigma). Images were created using a Leica TCS SP5 AOBS confocal inverted-base fluorescence microscope with HCX PL APO 40 × 0.75–1.25 oil immersion objective. The settings were adjusted using isotype control antibodies with similar concentrations. For each animal, 15 randomly selected images were made in order to create a representative image regarding the size of a ferret lung and analyzed for the integrated intensity in blue (DNA), green (DNA/histone-1 complexes), and magenta (MPO) channels using ImageJ 1.51q (National Institutes of Health, USA). Afterwards, the ratio of the green signal to the blue signal (%) was calculated for every single image. The mean of all images was used for the final statistical analysis.

### 2.12 Cell-free DNA, DNase-1 activity, and cathelicidin antimicrobial peptide ELISA

The amount of cell-free DNA in plasma and BALF was evaluated by a PicoGreen assay using Quant-iT™ PicoGreen™ dsDNA Assay-Kit (Thermo Fisher Scientific, USA). Briefly, 50 µL of plasma samples diluted with 50 µL of PicoGreen solution (according to manufacturer’s recommendation) was pipetted into a black, flat-bottom, cell culture 96-well plate (BRANDplates^®^, Merck, article number BR781608), incubated for 5 min at room temperature in the dark, and analyzed in a TECAN Spark plate reader (Tecan Austria GmbH, Austria).

The activity of DNase-1 was measured using DNase I Assay kit (Abcam ab234056) following the manufacturer’s instructions. Briefly, 25 µL of serum samples (diluted 1:2 with water), standards, and reaction mixes was pipetted in a flat base, nuclease-free, polysteren 96-well plate (Sarstedt, article number 83/82.1581.110), and the fluorescence kinetics was measured with Tecan Spark for 90 min at 37°C with interval time 1 min.

The concentration of ferret cathelicidin antimicrobial peptide (CAMP) was determined with a canine CAMP competition ELISA kit (Cat. Nr. E08C0333, BlueGene Biotech., Shanghai, China). Due to the unavailability of an ELISA kit targeting ferret CAMP, ferret CAMP isotype X2 sequence was compared in the Basic Local Alignment Search Tool (BLAST), and the highest similarity of 78% with *Canis lupus familiaris* was found. Serum samples were tested following the manufacturer’s instructions. Optical density of the samples was measured at 450 nm in a microplate spectrophotometer (Multiskan™ GO, Thermo Scientific).

### 2.13 Statistical analysis

The complete statistical analysis was performed in GraphPad Prism 9.0 (GraphPad Software, La Jolla, CA). For grouped data of more than two groups, one-way ANOVA with Tukey’s multiple comparisons test was used for normally distributed data, whereas Kruskal–Wallis test with Dunn’s multiple comparisons tests was performed for non-normally distributed data sets. Data sets comprising two groups were analyzed with paired (same animals before and after infection) or unpaired (different control and infected animals) two-tailed t-test (normally distributed data) or with Mann–Whitney test (non-normally distributed data). *p*-values below an alpha-level of 0.05 (*p* ≤ 0.05) were considered statistically significant. Virus titers from end-point dilution assay, histopathological results (nose, lung bronchi, alveoli, and vessel lesion scores), and results from cell-free DNA, DNase-1, and CAMP were analyzed with Pearson’s correlation. Furthermore, histopathological results expressing the amount of inflammatory lesions in the nose and lung (bronchi, alveoli, and vessel lesion scores) were correlated with the described NET markers and virological results.

## 3 Results

### 3.1 Clinical signs

The role of age in SARS-CoV-2-infected ferrets was assessed with the aim to characterize ferrets as a COVID-19 model. During the first 7 days after infection, seven of nine aged ferrets showed mild to moderate clinical signs. In contrast, only mild transient clinical signs were observed in two out of seven young ferrets at 1 to 2 dpi (38), while the other animals showed no obvious clinical signs. No animal had to be euthanized before the appointed time according to predetermined criteria for the humane endpoint.

### 3.2 Viral and RNA load in the upper and lower respiratory tract and other organs

**Figure 3:**
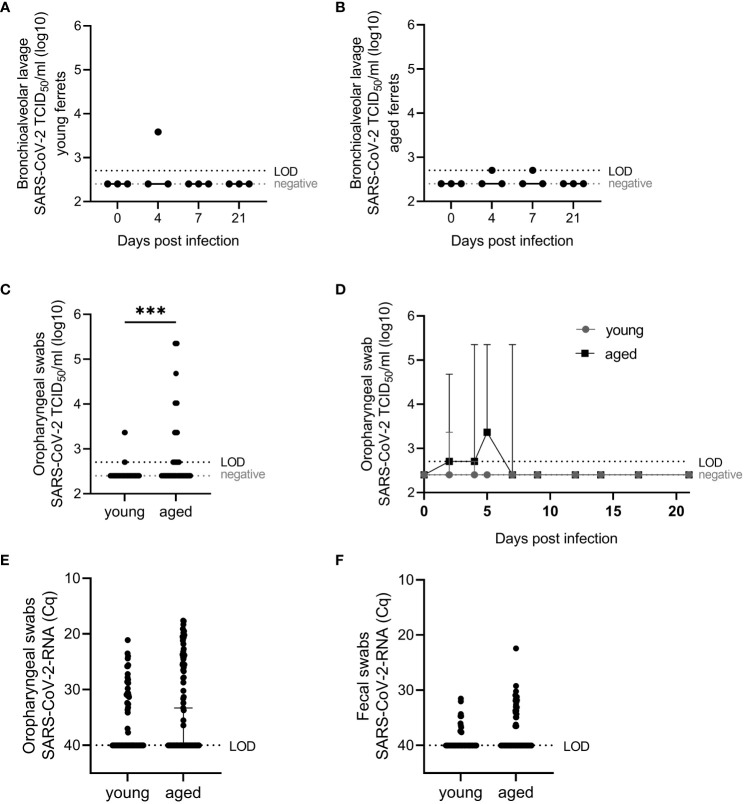
Virological results. SARS-CoV-2 viral infectious **(A–D)** and RNA **(E, F)** loads in young and aged ferrets quantified with end-point dilution assay (39) and SARS-CoV-2-specific real-time quantitative reverse transcription PCR for the detection of partial RNA-dependent RNA polymerase (RdRp) (38), respectively, in bronchioalveolar lavage fluid (BALF) and oropharyngeal and fecal swabs. SARS-CoV-2 RNA data from young ferrets were previously published (38) and used for statistical comparison of young and aged ferrets **(E, F)**. Whiskers represent median and 95% confidence interval **(A, B, D–F)** or median and error **(C)**. Kruskal–Wallis test **(A, B)** or Mann–Whitney test **(D–F)** were used for the non-normally distributed data to statistically analyze differences between viral loads in young and aged ferrets. TCID_50_/mL, tissue culture infectious dose 50%. Cq, quantification cycle value. LOD, limit of detection. ***p ≤ 0.001.

**Figure 4:**
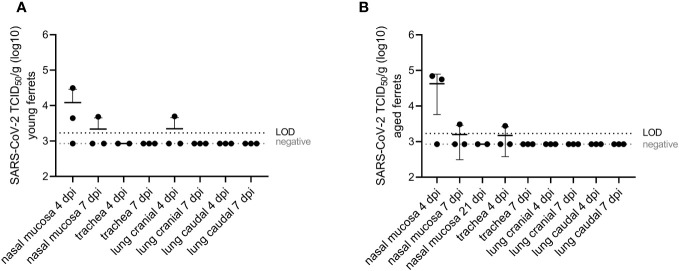
Virological results of respiratory tissue homogenates. Infectious SARS-CoV-2 quantified (TCID_50_/g) with end-point dilution assay (39) in respiratory tissue from young **(A)** and aged **(B)** ferrets. Whiskers represent mean and standard deviation. TCID_50_/g, tissue culture infectious dose 50% per gram.

The presence of SARS-CoV-2 RNA and infectious virus was evaluated in BALF, oropharyngeal and fecal swabs (**Figure 3**), upper and lower respiratory tract (RT) (**Figure 4**), and in a range of other organ tissues (**Figure 2**).

Viral RNA was detected in oropharyngeal swabs of all aged infected animals with a peak of virus load between 2 and 5 dpi. Infectious virus was generally recovered from the majority of tested samples between 2 and 7 dpi (see below and **Figure 3D**). In young compared with aged ferrets, we detected no statistically significant differences in the amount of virus RNA in oropharyngeal swabs and fecal swabs from all animals comparing PCR results (quantification cycle values, Cq) of all samples collected before infection until 21 dpi (**Figures 3E, F**). We recovered viable SARS-CoV-2 in four cases, twice at 2 dpi and twice at 4 dpi. We isolated significantly more infectious virus from oropharyngeal swabs in aged ferrets compared to young (**Figure 3C**).

Infectious SARS-CoV-2 in BALF from young and aged ferrets was detected in single ferrets up to 4×10^3^ TCID_50_/mL (young) and 5×10^2^ (aged) at 4 or 7 dpi (**Figures 3A, B**).

Furthermore, SARS-CoV-2 was isolated from oropharyngeal swabs of three young ferrets at 2 and/or 4 dpi (up to a titer of 2.3 × 10^3^ TCID_50_/mL, **Figure 3D**) and from eight of nine aged ferrets between 2 and 7 dpi (up to a titer of 2.3 × 10^5^ TCID_50_/mL each, **Figure 3D**). Statistically significant (p <0.05) higher infectious viral loads were found in oropharyngeal swabs of aged compared to young ferrets (**Figure 3D**). However, no statistically significant differences were found between SARS-CoV-2 RNA loads by age group in oropharyngeal and fecal swabs (**Figures 3E, F**).

Infectious SARS-CoV-2 was isolated from the nasal mucosa of each two (4 dpi) or one (7 dpi) of three young (≤3 ×10^4^ TCID_50_/g) and old (≤ 7×10^4^ TCID_50_/g) ferrets (**Figure 4**) and from the cranial lung lobe (one young ferret, 5 × 10^3^ TCID_50_/g) or from the trachea (one aged ferret, 3 × 10^3^ TCID_50_/g) at 4 dpi. No infectious virus was recovered from the lower RT of the aged group (**Figure 4**). No statistically significant differences were found in virus loads in the nasal mucosa between young and aged ferrets.

We found viral RNA in various tissue samples from aged ferrets, particularly high viral RNA loads in the upper and lower RT and associated tissues, including the nasal mucosa, trachea, cranial and caudal lung lobes, draining retropharyngeal lymph nodes, and tonsil with a peak at 4 dpi slowly decreasing until 21 dpi (**Figure 2**). Highest SARS-CoV-2 RNA loads (≤1×10^10^ copies/g) were detected in the nasal mucosa and draining retropharyngeal lymph nodes and tonsil up to 7 dpi in all six euthanatized aged animals and up to 21 dpi in one to two of three animals (**Figure 2**).

Several other organs were tested for viral RNA including the brain, third eye lid, olfactory bulb, heart, spleen, liver, gastrointestinal tract, kidney, and skeletal muscles. All tested organs of young ferrets were positive for viral RNA at 4 dpi, organs belonging to the RT still at 7 dpi, and only retropharyngeal and tracheobronchial lymph nodes positive at 21 dpi (38). All organs except for the kidney were positive in aged animals at 4 dpi, the majority of organs remained positive until 7 dpi, and only some positivity was found in the upper RT at 21 dpi (**Figure 2**).

### 3.3 Serology

Seroconversion (all three aged and two of three young ferrets) or a marked increase (one young ferret) in antibody levels until 21 dpi was detected with RBD-based iELISA in sera of all ferrets (**Supplementary Figures S1A–D**). In aged animals, a slight rise in antibody levels was already detected at 7 dpi with iELISA, but remained negative in the NT. In contrast to antibodies in young ferrets, antibodies (iELISA and NT) in aged animals were statistically significantly higher at 14 and/or 21 dpi compared to 0, 4, and 7 dpi **Supplementary Figures S1A–D**. Antibody levels and neutralizing titers were generally more homogenous in aged compared to young ferrets. However, no significant differences were found between age groups at 21 dpi (**Supplementary Figures S1E, F**).

### 3.4 Hematology

Statistical analysis of the hematological results with ordinary one-way ANOVA generally showed no statically significant (*p* < 0.05) differences before (−2 dpi) and at days 4, 7, and 21 after infection of young and aged ferrets, except for the percentual red blood cell distribution width (RDW) in young animals at 4 and 21 dpi. However, the RDW results in young animals were all within the range of the species-specific reference values (**Supplementary Figures S2A–E**).

### 3.5 Histopathology, immunohistochemistry, and scanning electron microscopy

**Figure 5:**
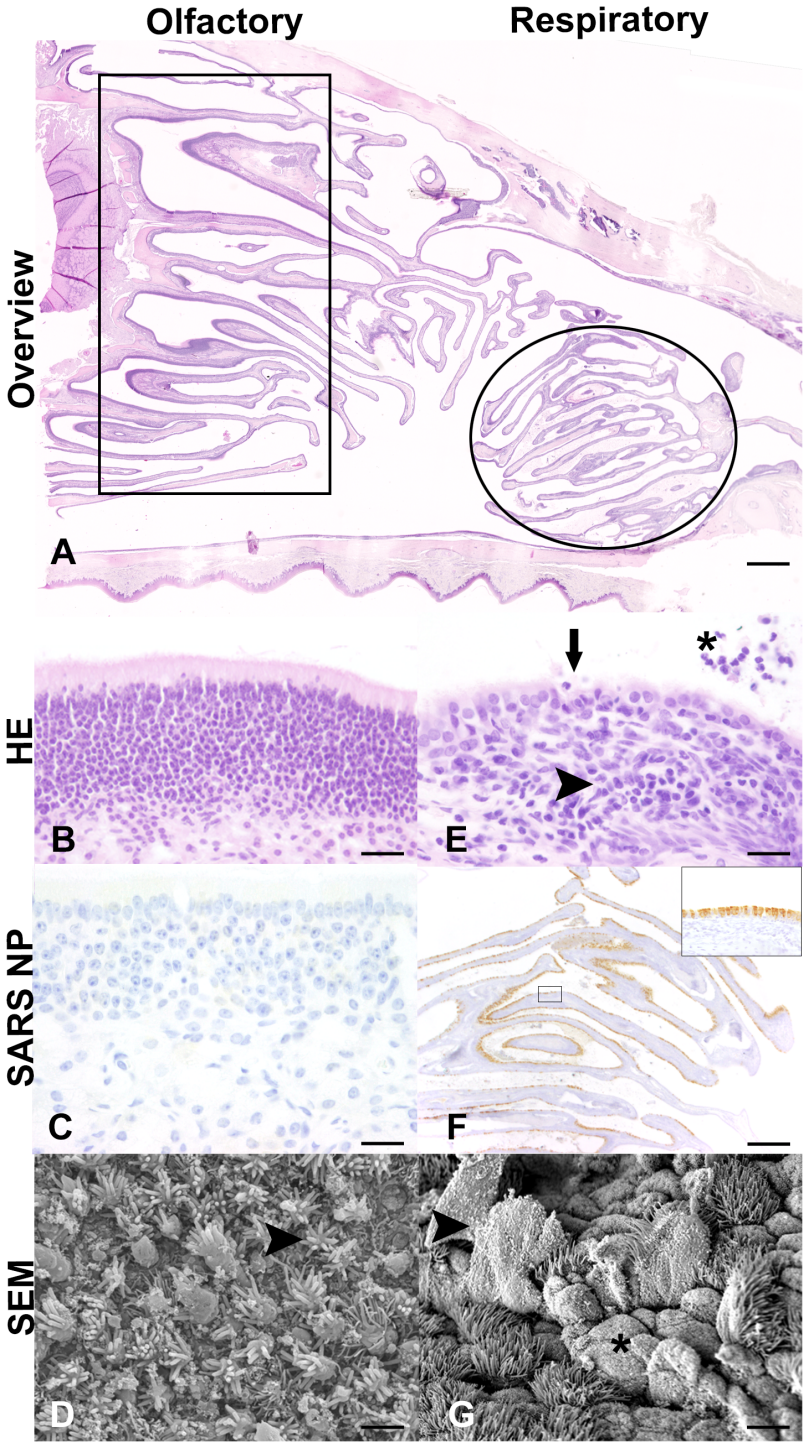
Histological **(A, B, E)**, immunohistochemical **(C, F)**, and scanning electron microscopic (SEM) **(D, G)** findings in the nasal cavity of SARS-CoV-2-infected aged ferrets at 4 days post-infection (dpi). **(A)** Light microscopic overview of the nasal cavity of a SARS-CoV-2-infected ferret at 4 dpi (F16). Rectangle: region of olfactory epithelium. Circle: region of rostral respiratory epithelium. Hematoxylin and eosin (HE) staining. Bar: 1 mm. **(B–D)** Olfactory epithelium. No inflammatory lesions (**B**, F16) or SARS-CoV-2 nucleoprotein (NP) (**C**, F17) detectable at 4 dpi. Bars: 20 μm. **(D)** SEM examination revealed normal olfactory epithelium characterized by dendritic bulbs armed with olfactory cilia (arrowhead) at 4 dpi (F16). Bar: 2 μm. **(E–G)** Respiratory epithelium. With inflammatory cells and lesions. **(E)** HE staining revealed nasal respiratory epithelium with subepithelial infiltration with lymphocytes, macrophages, plasma cells and few neutrophils (arrowhead), intraepithelial exocytosis and single cell desquamation (arrow), and intraluminal inflammatory cells and cellular debris (asterisk) (F16). Bar: 20 μm. **(F)** High amounts of viral NP detectable in multiciliated cells of the rostral respiratory epithelium (F16). Inset: Higher magnification of rectangle. SARS-CoV-2 NP immunohistochemistry using 3,3′-diaminobenzidine (DAB) as chromogen and Mayer’s hematoxylin as counterstaining. Bar: 20 μm. **(G)** Scanning electron microscopy (SEM) examination showed detachment and rupture of multiciliated cells (arrowhead) and ciliary loss (asterisk) in the rostral nasal cavity of a SARS-CoV-2-infected ferret at 4 dpi (F16). Bar: 2 μm.

**Figure 6:**
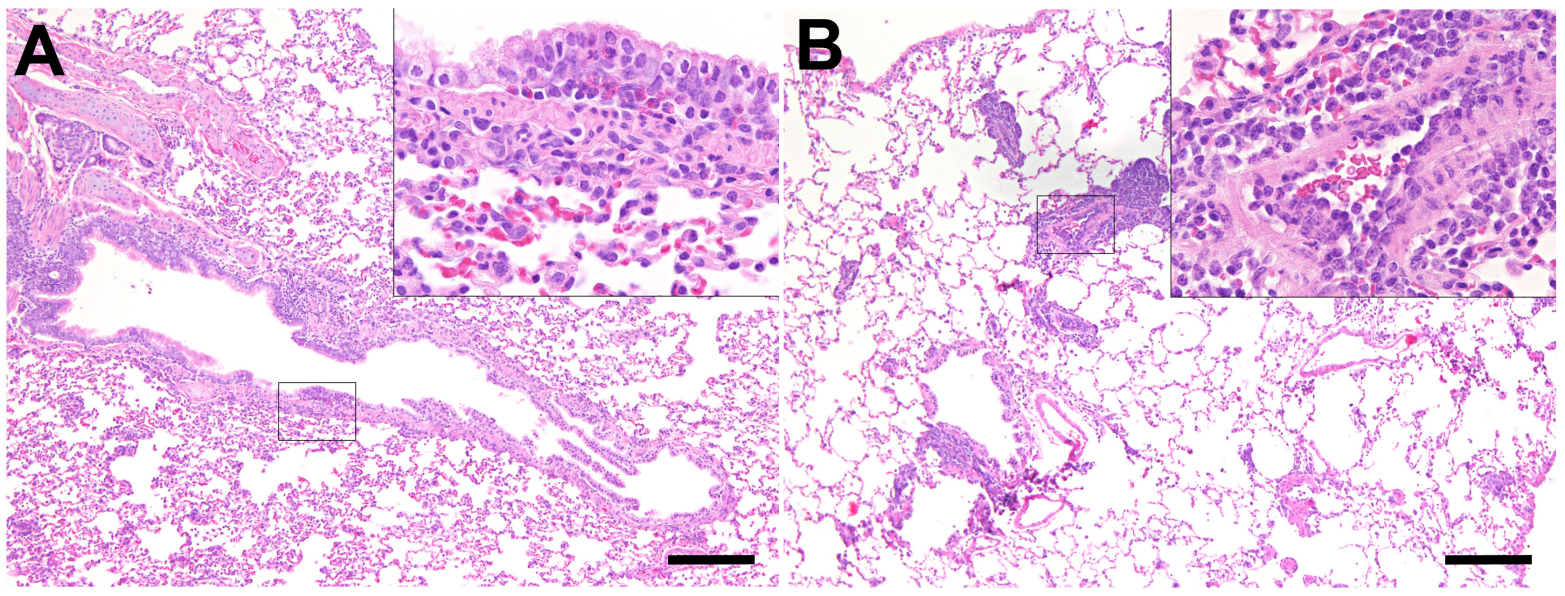
Inflammatory lesions in the lung of SARS-CoV-2-infected aged ferrets. Hematoxylin and eosin (HE) staining. **(A)** Bronchitis, lung, and SARS-CoV-2-infected ferret at 21 days post-infection (dpi, F22). There are several eosinophils and few lymphocytes in the bronchial mucosa. Inset: higher magnification. **(B)** Endothelialitis, lung, and SARS-CoV-2-infected ferret at 7 dpi (F20). The lung shows moderate multifocal vascular lesions and mild hyperplasia of bronchus-associated lymphoid tissue (BALT). Inset: Higher magnification of vascular lesions characterized by attachment of lymphocytes and macrophages to endothelial cells and subsequent migration into the subendothelial tissue. Bars = 200 µm (insets: bars = 50 µm).

No macroscopic lesions were detected on gross necropsy examination, except for an endogenous lipid pneumonia in one aged ferret at 7 dpi (F20). Inflammatory lesions in HE-stained respiratory organ sections from aged ferrets were examined (**Supplementary Table S3**). Control ferrets showed mild inflammatory lesions in the trachea and larynx (3/3 ferrets), lung (2/3 ferrets), and nose (1/3 ferrets). Histological, immunohistochemical, and scanning electron microscopic inflammatory cells and lesions in the nasal cavity were restricted to the respiratory epithelium in the rostral nasal cavity. In contrast, no inflammatory lesions were detected in the olfactory epithelium of aged SARS-CoV-2-infected ferrets at 4 dpi (**Figure 5**). The majority of infected ferrets had rhinitis with a mild to moderate inflammation with a peak at 7 dpi. The nasal mucosa was infiltrated by a moderate number of lymphocytes, macrophages, and plasma cells, and few neutrophils, which were also found in the lumen admixed with cellular debris. Inflammatory lesions and SARS-CoV-2-specific nucleoprotein (NP) were solely detected in the respiratory and not in the olfactory epithelium at all time points (**Supplementary Figures S3**, **S4**). Furthermore, all infected animals showed mild tracheitis (**Supplementary Figure S5**) and bronchitis (**Figure 6A**). The lungs of all infected ferrets except of one (F17) showed mild to moderate multifocal vascular lesions (endothelialitis) and a mild bronchitis (**Supplementary Table S3**, **Figure 6B**). In general, lesions in the upper and lower RT of young (38) and aged ferrets were more or less similar.

Similarly to our previous study (38), we found mild to moderate lesions in the extrarespiratory organs of control and infected ferrets, which are probably not related to the SARS-CoV-2 infection (**Supplementary Table S4**). Inflammatory lesions in the heart, mesenterial lymph node, gastrointestinal tract, pancreas, liver, gall bladder, kidney, adrenal gland, thyroid gland of control and SARS-CoV-2-infected ferrets are shown in **Supplementary Figure S6**.

**Figure 7:**
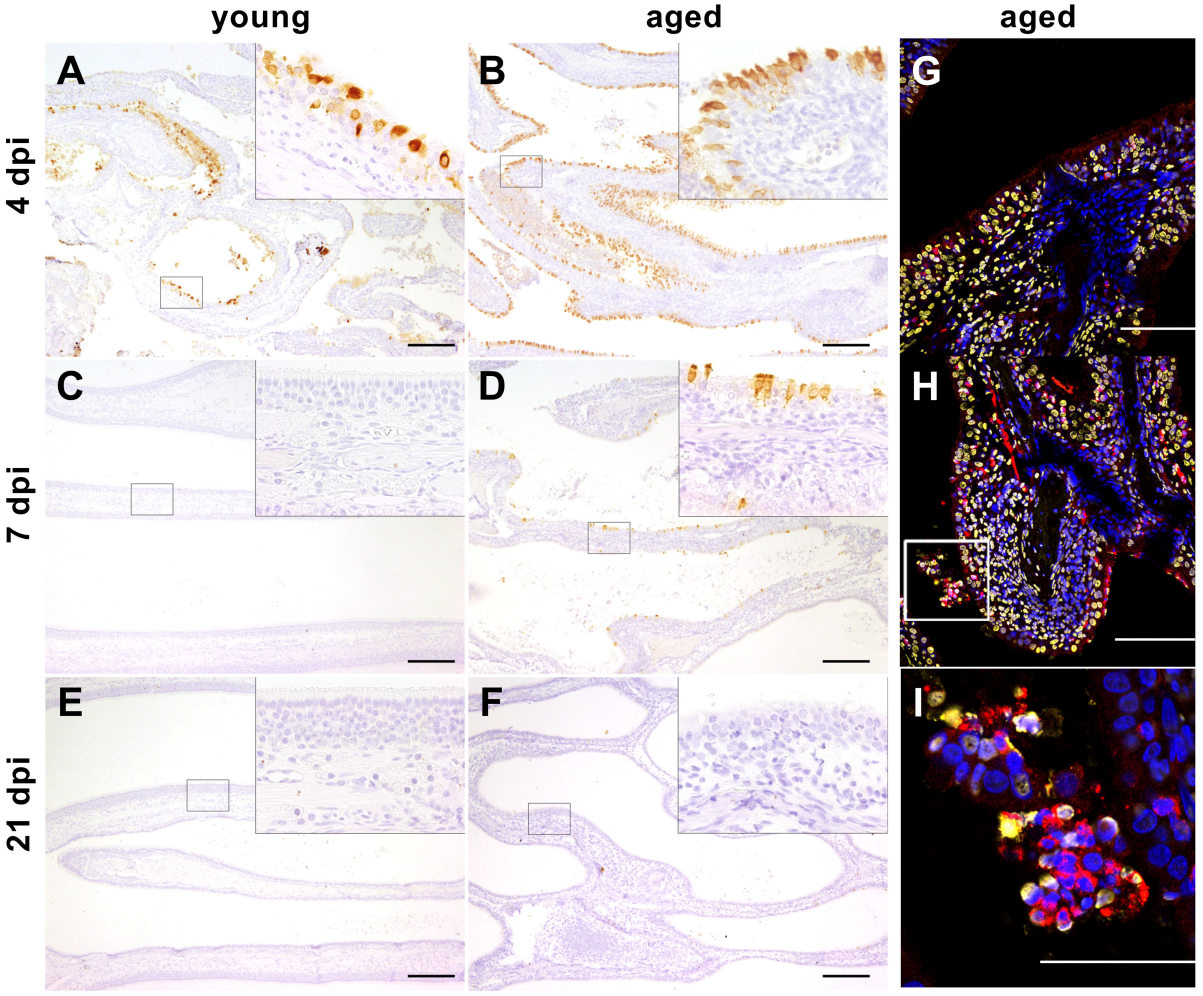
**(A–F)** Immunohistochemistry. Severe acute respiratory syndrome coronavirus (SARS-CoV)-2 infection, nose, young, and aged ferrets, 4, 7, and 21 days post-infection (dpi). Positive signal (brown) for SARS-CoV-2 nucleoprotein (NP) detected in the ciliated epithelial cells of the respiratory mucosa at 4 dpi in young ferrets (**A**, F5) and at 4 and 7 dpi in aged ferrets (**B**, F16; **D**, F19). No positive signal for SARS-CoV-2 NP present in the nose at 7 and 21 dpi in young ferrets (**C**, F8; **E**, F12) and at 21 dpi in aged ferrets (**F**, F23). Higher magnifications show details of the areas enclosed by the rectangles. Bars = 200 µm (insets: bars = 50 µm). **(G–I)** Immunofluorescence staining of the nasal conchae from SARS-CoV-2-infected aged ferrets at 4 dpi with antibodies against myeloperoxidase (MPO, red) and DNA/histone-1 complexes (yellow). Nuclei are stained with Hoechst 33342 (blue). **(G)** F17. **(H)** F16. **(I)** F16 (inset of **Figure 7H**) magnification of a rectangular in image **(H)**. Intraluminal accumulation of desquamated cells and neutrophils with adjacent MPO and DNA/histone-1 signal. Bars = 100 µm **(G, H)** or 50 µm **(I)**.

SARS-CoV-2-specific NP was detected in the ciliated epithelial cells of the respiratory mucosa of all young ferrets sacrificed at 4 dpi and all aged ferrets at 4 and 7 dpi (**Figures 7A–F**). No positive signal was found in the lungs of young and aged ferrets (**Supplementary Figure S4**). Moreover, we detected FRSCV in a mesenterial lymph node of an infected animal (F16) sacrificed at 4 dpi and in an adrenal gland of a control animal (F14) (**Supplementary Figure S8**).

### 3.6 NET analysis

#### 3.6.1 NET and SARS-CoV-2 antigen detection in the nasal epithelium

Activated neutrophils with attached MPO and DNA/histone-1 signals were detected in aged ferrets at 4 dpi in the respiratory epithelium of the intraluminal space of the nasal cavity, indicating NET formation (**Figures 7G–I**) similar as in young ferrets as previously described (38). Furthermore, in accordance with our previous study of young ferrets (38), immunohistochemical analysis revealed SARS-CoV-2 antigen in the respiratory epithelium of the nasal cavity at 4 dpi (young and aged) and 7 dpi (aged ferrets) (**Figures 7A–F**) (see details in *Section 3.5 Histopathology and immunohistochemistry*) and an association with NET formation at 4 dpi (**Figures 7G–I**: aged (38);: young). Since quantitative analysis in nasal conchae is extremely challenging due to high occurrence of artifacts and background signals, further assays focused on quantification of NET marker in the lung, serum, and BALF.

#### 3.6.2 NET detection in lung biopsies

**Figure 8:**
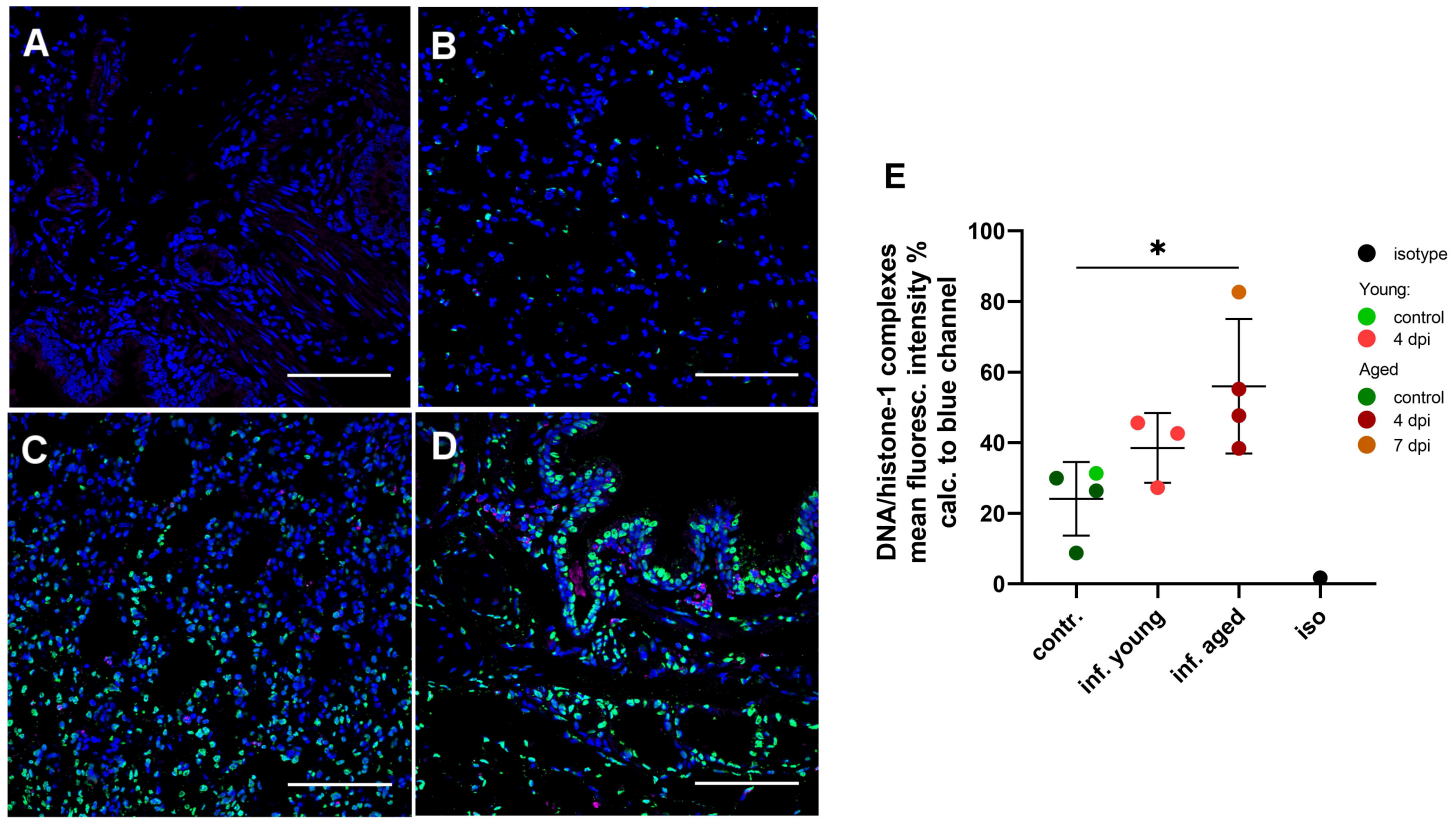
Detection and quantification of NET formation in lung tissue sections in young and aged ferrets with double immune-labeling targeting the DNA/histone-1 complexes (green) and myeloperoxidase (magenta). Nuclei were stained with Hoechst 33342 (blue). **(A)** Isotype control. **(B)** Control non-infected aged ferret F13. **(C)** Infected young ferret euthanized 4 days post-infection (dpi) (F4). **(D)** Infected aged ferret euthanized 4 days post-infection (dpi) (F16). Bars = 100 µm **(A–D)**. **(E)** Quantification of DNA/histone-1 complexes signal in lungs with confocal microscopy. Mean fluorescence intensity (%) of the green channel (DNA/histone-1) was calculated to the blue channel (nuclei). Each data point represent mean intensity of 15 images made for each sample. Contr.: aged (dark green) and young (light green) control ferrets euthanized 2 days before infection. Inf. young (light red): animals euthanized 4 dpi. Inf. aged: infected animals euthanized 4 dpi (dark red) and 7 dpi (dark orange). Iso: isotype control. Data were analyzed with an unpaired two-tailed one-way ANOVA test. Whiskers represent mean and standard deviation. *p ≤ 0.05.

Using confocal fluorescence microscopy, we confirmed the presence of NETs in lung tissue sections from infected young and aged ferrets stained with antibodies targeting DNA/histone-1 complexes (green) (**Figure 8**). Quantification of the green channel for DNA/histone-1 complexes relative to nuclear DNA revealed a significant increase in the lungs of four selected aged infected ferrets at 4 and 7 dpi compared to four selected control animals (**Figure 8E**), confirming enhanced NET formation in lungs of infected aged ferrets.

#### 3.6.3 NET markers in serum and BALF

**Figure 9:**
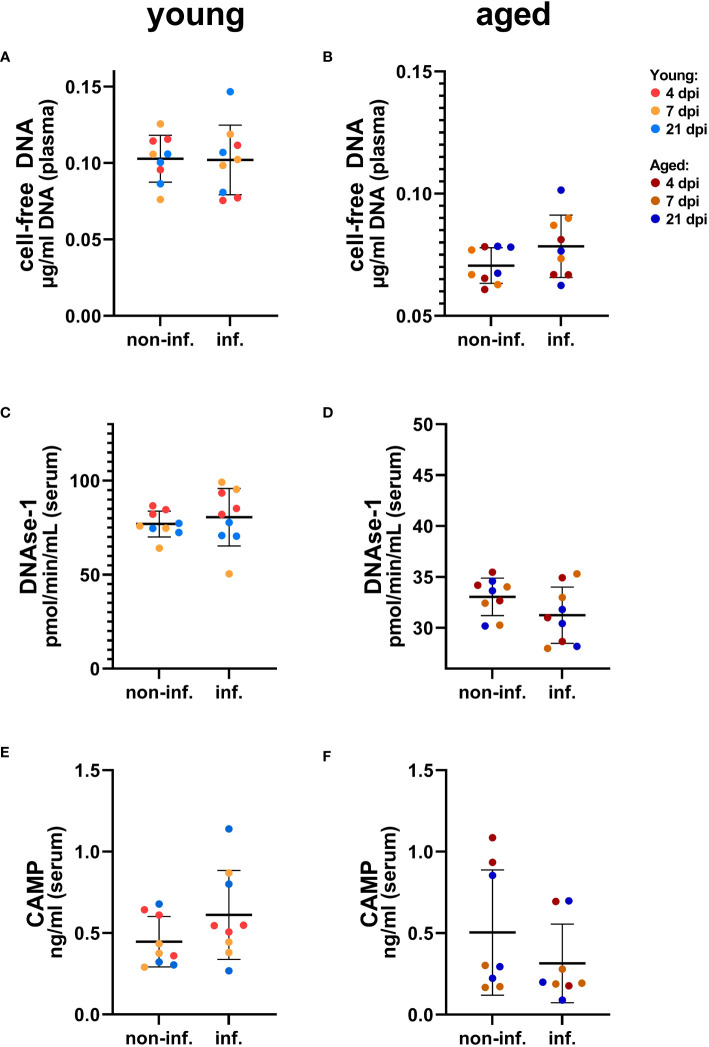
Detection of NET markers in serum of young **(A, C, E)** and aged **(B, D, F)** ferrets. **(A)** Cell-free DNA in young ferrets. **(B)** Cell-free DNA in aged ferrets. **(C)** DNase-1 activity in young ferrets. **(D)** DNase-1 activity in aged. **(E)** Cathelicidin antimicrobial peptide (CAMP) ELISA in young ferrets. **(F)** CAMP ELISA in aged. Non-inf.: serum from all animals before infection. Inf.: serum from all the same animals after infection. Data were analyzed with a paired two-tailed t-test. Whiskers represent mean and standard deviation.

To analyze selected NET markers in the serum or plasma of young and aged ferrets, we performed the combination of three assays detecting the cell-free DNA, the activity of DNase-1, and concentration of CAMP. In serum from young and aged ferrets, we found no differences in any of the three assays between samples taken from the same animals before and after infection (**Figure 9**, **Supplementary Figure S9**). The concentrations of CAMP in serum from young and aged ferrets before and after infection were similar (**Figure 9**).

**Figure 10:**
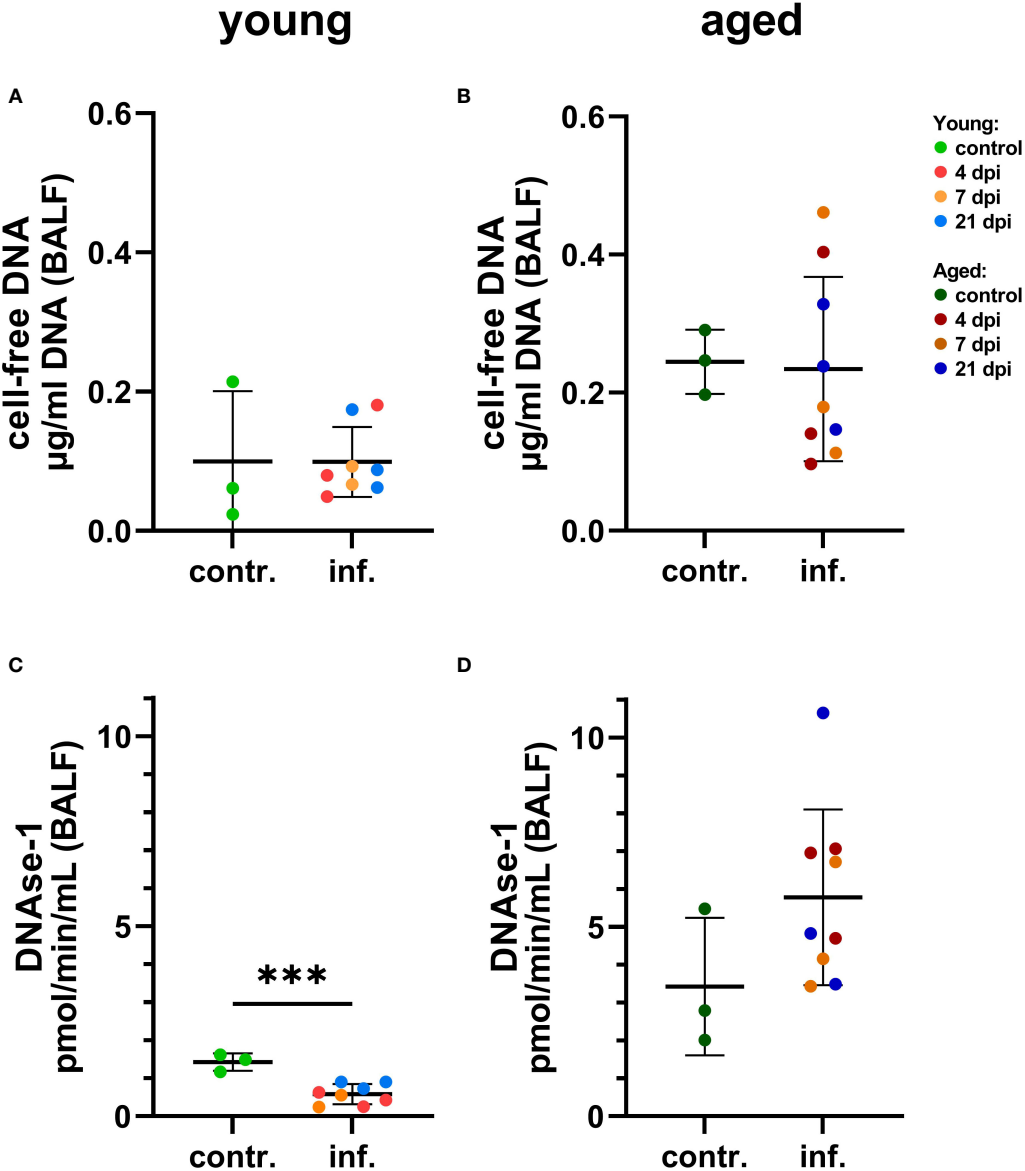
Detection of NET markers in bronchoalveolar lavage fluid (BALF) of young **(A, C)** and aged **(B, C)** ferrets. **(A)** Cell-free DNA in young ferrets. **(B)** Cell-free DNA in aged ferrets. **(C)** DNase-1 activity in young. **(D)** DNase-1 activity in aged. Contr.: BALF from three control animals euthanized 2 days before infection. Data were analyzed with an unpaired two-tailed t-test. Whiskers represent mean and standard deviation. ***p ≤ 0.001.

Interestingly, a significantly lower DNAse-1 activity was found in BALF of young infected compared to young control ferrets considering individual days (**Figure 10**, **Supplementary Figure S10**), while no significant differences were found for young infected versus control young or for aged infected compared to aged control ferrets (although DNase-1 activity was markedly increased by SARS-CoV-2 infection) (**Figure 10**, **Supplementary Figure S10**). We also investigated the influence of age on both markers in BALF samples and detected more cell-free DNA and higher DNase-1 activity in aged compared to young infected ferrets (**Supplementary Figure S11**). This contrasts the significantly decreased cell-free DNA and DNase-1 activity found in plasma or serum of aged ferrets if compared to young ferrets (**Supplementary Figure S11**), although no significant differences were found between infected and control ferrets independent from the age group (**Supplementary Figure S11**).

Comparing control groups, no significant difference in cell-free DNA and DNase-1 activity was found in BALF between young and aged control ferrets (**Supplementary Figure S11**), but significantly lower amounts of cell-free DNA and DNAse-1 were found in plasma or serum of aged compared to young control ferrets (**Supplementary Figure S12**).

#### 3.6.4 Correlation analysis of NETs with additional clinical markers

**Figure 11:**
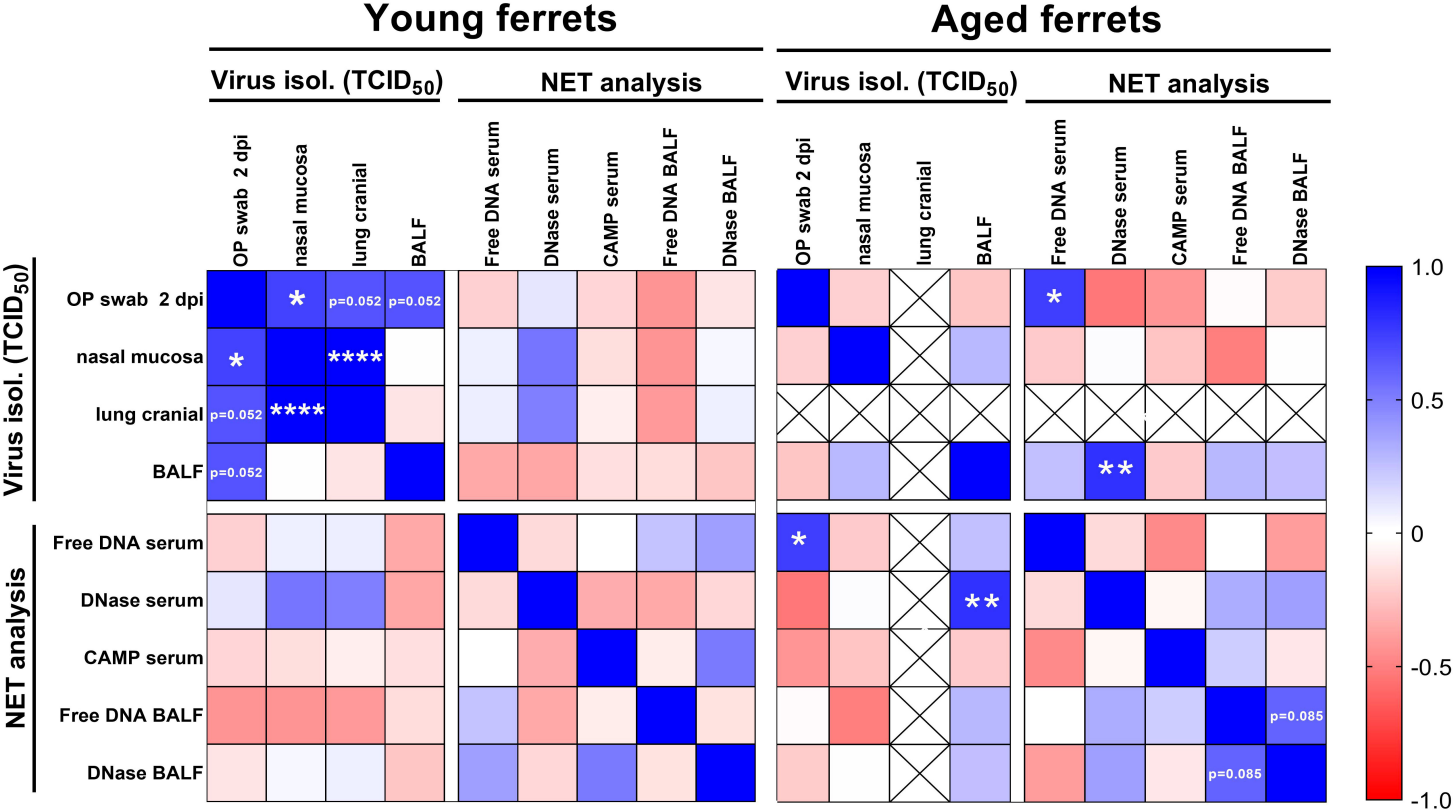
Correlation matrix of virus titers from end-point dilution assay and results from NET marker analysis. Virus titers from oropharyngeal (OP) swabs from 2 days post-infection (dpi), nasal mucosa, cranial lung, and bronchoalveolar lavage fluid (BALF) were included in the analysis. Cell-free DNA, DNase-1 activity, and CAMP concentration in serum and cell-free DNA and DNase-1 activity in BALF results were used for the correlation. Data were analyzed with parametric Pearson’s two-tailed correlation with 95% confidence interval. Only data from young and aged infected animals (4–21 dpi) were included in the analysis. Crossed out fields show data without correlation analysis (no virus was isolated from the cranial lung lobe of aged ferrets). Results with p-values between 0.05 and 0.1 are directly shown in the figure. *p ≤ 0.05. **p ≤ 0.01. ****p ≤ 0.0001.

A correlation analysis was performed to reveal if there is any correlation between selected NET markers in serum and BALF and virus titers in oropharyngeal swabs at 2 dpi, BALF, nasal mucosa, and cranial lung. In young ferrets, we found no correlation between virus titers, cell-free DNA, DNase-1, and CAMP using Pearson’s correlation (**Figure 11**). However, significantly positive correlations were found between virus titers in the nasal mucosa and oropharyngeal swabs at 2 dpi or cranial lung lobe. Additionally, positive correlations of viral titers between oropharyngeal swabs and BALF or oropharyngeal swabs and cranial lung lobe (both p=0.052) were detected in young ferrets. In the serum of aged ferrets, the amount of cell-free DNA significantly correlated with the virus titers found in the oropharyngeal swabs (p=0.022), and the level of DNase-1 activity strongly correlated with the virus titer found in the BALF (p=0.01). Moreover, in the BALF of aged ferrets, we detected a positive correlation between the DNase-1 activity and the amount of cell-free DNA (p=0.085). Interestingly, pooling the data of young and aged ferrets together significantly strengthened this correlation (p=0.001), while other correlations, except of the one between oropharyngeal swab at 2 dpi and cell-free DNA (p=0.05), were lost (**Supplementary Figure S13**). Correlation analysis of histopathological results (nose, lung bronchi, alveoli, and vessel lesion scores) did not show significant correlations with NET markers or virological results, except for DNase-1 activity in BALF and scores in the nasal tissue in the pooled data set (**Supplementary Figure S13**).

## 4 Discussion

In this study, young and aged female ferrets were assessed as an animal model resembling COVID-19 in humans with a focus on the viral loads, immune response, and formation of NETs. Previous studies, for example by the groups of Au et al. (1) and Kim et al. (36) have focused on other immune parameters such as hematology, clinical chemistry (1), or on transcriptional profiles of immune-related genes in the lungs of SARS-CoV-2-infected young and aged ferrets (36).

Both age groups were intratracheally (i.t.) infected with 10^6^ TCID_50_/mL SARS-CoV-2, which is a high dose recommended for SARS-CoV-2 infection in ferrets (15, 43). The majority of other studies chose intranasal (i.n.) infection route (1, 6, 7, 9–13, 15–17, 19, 43) or directly compared the i.n. to i.t. infection (8, 14) and came to a conclusion that the i.n. route leads to more efficient infection (14). Our literature research supports the theory that young age and a low-infection dose favor asymptomatic and ineffective infection (15, 43). Here, we demonstrate a successful intratracheal SARS-CoV-2 infection in both the upper and lower RT of all ferrets with an age-dependent effect.

Previously, young ferrets remained asymptomatic or showed very mild signs of disease (38), while in aged ferrets in the present study, mild to moderate clinical signs, including respiratory signs, were observed. These results are in line with the studies of McAuley et al. (44) and Kim et al. (12, 16) that described very similar clinical signs in female ferrets of comparable age groups as in this study (12, 16). Interestingly, the scientific community agrees on young ferrets (approximately under the age of 12 months) infected with SARS-CoV-2 to be mainly asymptomatic, but the clinical picture in aged ferrets highly differs. For instance, there are studies describing a complete lack of any clinical signs and no infection in lungs in adult ferrets (18–48 months) infected with a high dose (10^6^–10^7^ TCID_50_/mL) (14, 15). On the other hand, these ferrets were younger (1.5–2 years) than the aged ferrets in our study and the study of Kim et al. (16) (> 3 years). Furthermore, co-infections might play a role in disease expression (see below).

In humans, risk factors for the development of ARDS in COVID-19 disease include age, comorbidities, fever >39°C, and altered blood chemical and hematological values (e.g., increased lactate dehydrogenase, significantly higher neutrophil granulocyte, or lower lymphocyte counts) (45, 46). Although age was found to be a risk factor for aged ferrets to develop respiratory signs, SARS-CoV-2 infection showed no obvious effect on hematological parameters regardless of the age (**Supplementary Figures S2A–E**).

The serological results confirmed successful SARS-CoV-2 infection. All aged ferrets seroconverted at 14 dpi and stayed positive until day 21 (iELISA and NT). Two out of three young ferrets seroconverted at 14 dpi (iELISA) and 21 dpi (NT). Nevertheless, the one young seronegative ferret showed a considerable increase in antibody levels (iELISA) up to slightly under the detection limit of the iELISA at 14 and 21 dpi (**Supplementary Figure S1**). Similarly, Au et al. (1) found that one-fourth of young ferrets (4 months old) had neutralizing antibodies at 14 dpi (the day of euthanasia) and suggested that young age had an influence on the low seroconversion rate. Furthermore, a more distinct or significantly higher increase in SARS-CoV-2-specific antibody levels was reported in adult humans compared to children (47).

Viral RNA was detected in the nasal mucosa, oropharyngeal swabs, BALF, trachea, and lungs in young (38) and aged ferrets in the present study (**Figures 2**, **3**) at 4 dpi and 7 dpi. Considerably more tissue samples were positive at 7 dpi in aged (**Figure 2**) compared to young ferrets (38). The majority of other studies also detected viral RNA in the upper RT (1, 6–14, 17, 19, 43), but occasionally, no viral RNA was detected in the lungs (8, 17). While the presence of RNA in the respiratory tissues is already well described, the knowledge about SARS-CoV-2 dissemination into other tissues is often missing and further investigation is recommended (10). Therefore, extrarespiratory tissues were tested for SARS-CoV-2 RNA, and a similar trend of a prolonged and stronger distribution into the extrarespiratory and respiratory organs of aged ferrets until 7 dpi and in retropharyngeal lymph node and tonsils even until 21 dpi (**Figure 2**) was observed compared to young ferrets of our previous study (38). No SARS-CoV-2 RNA was found in the kidney of aged ferrets, which is in line with other studies (17). However, in our previous study of SARS-CoV-2 infection in young ferrets, we found a broader distribution of SARS-CoV-2 RNA and generally higher viral RNA loads in all tested organ samples in one individual young ferret at 4 dpi compared to the other eight young ferrets in the study (38).

In addition to SARS-CoV-2 infection, we found FRSCV-antigen in a mesenterial lymph node of one SARS-CoV-2-infected ferret and in the adrenal gland of one control ferret (**Supplementary Figure S8**). FRSCV infection was most likely subclinical due to the lack of characteristic gross and histological lesions and the absence of FRSCV antigen in other organ systems. FRSCV might have impaired the immune system. However, the FRSCV antigen was detected in both a control and an infected animal and independent from clinical, hematological, or histopathological scoring results (**Supplementary Table S4**). Therefore, we assume that this had no or a negligible influence on our data or the disease outcome.

Infectious virus was isolated from the nasal mucosa at 4 and 7 dpi in young and aged ferrets (**Figure 4**) and from oropharyngeal swabs at 2 or 4 dpi (three of nine young and eight of nine aged ferrets) until 5 or 7 dpi (five and two aged ferrets, respectively) with a prolonged shedding until 7 dpi and statistically significant higher titers in aged ferrets (**Figure 3**). In contrast to the results of the nasal mucosa and oropharyngeal swab samples, infectious SARS-CoV-2 was only recovered from BALF of one young and two aged ferrets, and from the cranial lung lobe of another young ferret (**Figures 3**, **4**). This could be explained by a relatively rapid inactivation of viral infectivity of clinical samples (6), particularly in samples with low viral or RNA loads (**Figures 2**–**4**). Other research groups also recovered SARS-CoV-2 from the upper RT, but the isolation of infectious virus from the lungs, despite of (high) RNA loads detected in the lungs, was often challenging (1, 6, 14). Furthermore, in studies using very young ferrets (3–4 months old), no respiratory signs, no infectious virus, and no viral RNA in lungs were found, independently of the infectious dose and route (7, 8).

Signs of inflammation in the RT of aged ferrets, including rhinitis restricted to the respiratory mucosa, tracheitis, laryngitis, and bronchitis with associated epithelial damage were found in the infected animals from 4 dpi until 21 dpi (**Figures 6**, **7**; **Supplementary Figure S3**, **S5**, **S7**), which were similar to lesions previously reported in young ferrets intratracheally infected with SARS-CoV-2 (38). Some minor lesions were also found in the control group, which could be explained by the age of the animals.

SARS-CoV-2-specific antigen was detected in the nasal conchae of young (4 dpi) (38) and aged ferrets (4 and 7 dpi) (**Figures 5F**, **7**), but surprisingly, despite the presence of viral RNA in the lungs of all aged ferrets at 4 dpi and 7 dpi (**Figure 2**) and the recovery of infectious virus from BALF of two aged ferrets at 4 dpi or 7 dpi (**Figure 3**), no antigen was detected in the lungs (**Figure 6**). Similar observations were described (10), despite a positive SARS-CoV-2 isolation from lungs.

Summarizing the clinical, virological, and histopathological data, ferrets were confirmed to be a fitting model for SARS-CoV-2 infection, depending on the research objective. However, little is known about the formation and impact of NETs in ferrets (38, 48).

The close association of SARS-CoV-2 antigen in the respiratory mucosa (**Figure 5**) with activated neutrophils and NET markers in the nasal conchae (**Figure 7**) and oropharyngeal shedding (**Figure 3**) and virus isolation from the nasal mucosa (**Figure 4**), but absence of virus antigen in the lungs (**Supplementary Figure S7**), confirm the upper RT (particularly the respiratory epithelium) as the main source of virus replication of SARS-CoV-2 in aged ferrets as previously described in young ferrets (38).

Despite the absence of SARS-CoV-2 nucleocapsid protein in the lungs, we also investigated lung biopsies for the presence of NETs, as they are not always formed only by activated neutrophils as a response to the SARS-CoV-2 virus itself. In fact, NET formation can also be induced by several immune factors independent of an infection, e.g., by the proinflammatory mediators interleukin (IL)-8 and IL-1β, autoantibody overexpression, or activated platelets (49–51). For example, the study of Wu et al. (52) found low virus loads but a high concentration of NETs and thromboses in lung biopsies from patients who died of severe COVID-19 (52).

To test whether NETs could contribute to the pathogenesis of SARS-CoV-2-infections in ferrets, a screening for NET markers using serum or plasma and BALF was performed. As NETs mainly consist of DNA, the amount of cell-free DNA was evaluated by a PicoGreen assay, but did not reveal any differences between infected and control animals. Hereby, it needs to be mentioned that cell-free DNA in body fluids might also originate from sources other than neutrophils (53) and also especially by necrotic cells; thus, cell-free DNA as marker is not NET specific. Host nucleases are crucial for maintaining the balance between NET formation and elimination and hence for preventing detrimental accumulation of NETs. Therefore, nuclease production is often used as additional indirect marker for NET formation (54, 55). In our study, the infected aged animals show a clear tendency for more nuclease activity compared to uninfected animals in BALF, but not for serum values. In contrast, DNAse-1 activity in serum of aged ferrets was significantly lower compared to that in young ferrets, but no significant differences were found between infected and control ferrets independent from the age group (**Supplementary Figure S12**). In accordance with these data, immunofluorescence microscopy revealed significantly more NETs in lungs of aged compared to young ferrets. Remarkably, we observed a significantly positive correlation between cell-free DNA in serum and virus isolation from oropharyngeal swabs and a significantly positive correlation of the DNase-1 activity in serum with virus isolation in BALF in aged ferrets (**Figure 11**, **Supplementary Figure S13**), pointing towards a partial COVID-19 disease-associated formation of NETs or release of cell-free DNA from necrotic cells.

In good correlation to these data, we detected infiltrating neutrophils into the lung tissue of SARS-CoV-2 infected young and aged ferrets and the release of NETs with a stronger phenotype in the aged animals using confocal fluorescence microscopy. No NET markers were observed in the lungs of control ferrets (**Figure 8B**). By quantification of the mean fluorescence intensity of the green channel (DNA/histone-1) as marker for NETs relative to the blue channel (nuclei), a statistically significant or considerable increase in NETs in the aged and young ferrets, respectively, euthanized at 4 dpi compared to the uninfected controls were detected (**Figure 8E**).

However, since no clear correlations of all tested NET markers in BALF or serum were detectable among each other or regarding virological results, only a minor unclear role of NETs might be summarized.

In COVID-19 patients, increased levels of NET markers circulating in serum were described (21, 31, 56). While cell-free DNA is increased in serum or plasma of diseased compared to control individuals (21, 57), DNase-1 activity is sex and age dependent and was recently detected increased in female but decreased in male individuals (31). Furthermore, reduced DNAase-1 associated with increased cathelicidin levels in elderly men was identified as risk factor for NET-associated thrombosis (31). Since in our study we used only female ferrets, this phenomenon in men could not be confirmed in our study with ferrets. In this study, we did not see expected significant differences in DNase-1 activity or cathelicidin levels, neither of cell-free DNA in the serum nor in BALF of controls and infected young or aged female ferrets. Although we detected a statistically significant decrease in DNase-1 activity in BALF of young infected ferrets comparing the control group with the infected groups by euthanasia day (**Supplementary Figure S10**), no statistically significant difference was found comparing the control group with all infected young animals (**Supplementary Figure S11**). Low nuclease activity was also identified in human blood or BALF that was linked to a severe form of SARS-CoV-2 infection and other significantly elevated NET markers or to SARS-CoV-2 infection in men (31, 58, 59). Since we compared nuclease activity in young and aged female ferrets, comparative studies about NET formation in male ferrets would be required in the future. On the other hand, no statistically significant differences between 4- and 10-month-old females and males experimentally infected with SARS-CoV-2 were reported for the parameters clinical signs and SARS-CoV-2 or RNA shedding by Au et al. and Monchatre-Leroy et al. (1, 17), or for SARS-CoV-2-RNA tissue distribution, hematological, and clinical chemistry parameters (1). Furthermore, no obvious clinical signs were observed in young (9–10 month old) and aged (36–48 months old) male ferrets by the group of van de Ven et al. (14). According to our results and the groups of Au et al., Monchatre-Leroy et al. and van de Ven et al. (1, 14, 17), we speculate that sex does not play a major role in NET formation in ferrets after SARS-CoV-2 infection.

Due to the ferret’s lifespan of 5–10 years (60), our 3–3.5-year-old animals correspond to mid-aged adults that usually, if no other co-morbidities are present, suffer from a mild to moderate form of COVID-19, as shown in our aged ferret model. Unfortunately, NET formation in the context of COVID-19 is in the literature described in patients suffering from severe or even critical stage of the disease, which makes our data hard to compare. Thus, young and aged ferrets are not proper valid models for severe stages of diseases associated with massive detrimental NET formation. Although further research on elderly ferrets is recommended, old ferrets may suffer from other co-morbidities or co-infections that might impair the immune system, e.g., neoplasia or other infectious diseases like Aleutian disease, which might make the association with the disease outcome in humans difficult (60). In general, commercially available ferrets are tested specific pathogen-free against certain pathogens. Most importantly, they are tested free for other bacterial and viral pathogens that are known to aggravate clinical signs, such as *Carnivore amdoparvovirus 1* (Aleutian disease), influenza virus, *Bordetella bronchiseptica*, and *Streptococcus pneumoniae*. However, co-infections with other pathogens, such as FRSCV (**Supplementary Figure S5**, see earlier), ferret enteric coronaviruses (**Supplementary Table S2**) (35), and ferret hepatitis E virus (FRHEV) (61), or other underlying health issues, are common in ferrets (35) and might in certain individuals play a role in disease expression after SARS-CoV-2 infection. For example, periportal infiltration with mononuclear cells in the liver as found in the present study in aged ferrets (**Supplementary Figure S6E**) has been associated with co-infections including FRHEV, SARS-CoV-2, ferret enteric coronavirus, intestinal parasites, or *Helicobacter mustelae*. Interestingly, large changes in the microbiome composition of the upper respiratory tract over time and between individuals were described in a longitudinal study in ferrets and humans after infection with influenza virus, which might influence disease expression (62). Hence, thorough investigation of the possible disease etiology of clinical signs or histopathological alterations that are not pathognomonic might help to understand disease expression in ferrets in the future (61).

Golden Syrian hamsters were found to be a suitable animal model for SARS-CoV-2 due to their high susceptibility and transmissibility and even resemble humans in SARS-CoV-2 pathogenesis associated with NETs and vasculitis (24, 63–65). However, despite more consistent weight loss and lung pathology in aged hamsters, the virus replication in the upper and lower RT was independent of the age (66). Additionally, the viral clearance in the respiratory tract was observed generally earlier in hamsters compared to infection in humans, which might be a limitation in a long-term pathogenesis research (65, 67).

Despite the increased NET formation in infected lungs of aged ferrets, the animals did not show a strong phenotype for NETs, and thus, they are of limited use to study SARS-CoV-2 pathogenesis associated with NETs in humans. However, comparing the young and aged ferret model, significantly more infectious virus and viral RNA and prolonged shedding were found in oropharyngeal swabs and the upper respiratory tract of aged ferrets. Together with more severe clinical signs and the lung pathology, our data again confirm aged ferrets as a relevant model for age-dependent COVID-19 pathogenesis.

## Data availability statement

The original contributions presented in the study are included in the article/**Supplementary Material**. Further inquiries can be directed to the corresponding authors. In this study, we compare new data from aged ferrets with data from young ferrets that were already published by our research groups for comparison, focusing on statistical analysis (38). The reutilization of published data are referred to the published manuscript (38) in the respective section and captions of the manuscript. Furthermore, we provide additional data from the young ferrets that were not published previously.

## Ethics statement

The animal study was approved by Niedersächsisches Landesamt für Verbraucherschutz- und Lebensmittelsicherheit (LAVES), Oldenburg, Germany, permission number: 33.19-42502-04-20/3402. The study was conducted in accordance with the local legislation and institutional requirements.

## Author contributions

VP: Data curation, Formal Analysis, Investigation, Methodology, Visualization, Writing – original draft. IG: Conceptualization, Data curation, Formal Analysis, Investigation, Methodology, Validation, Visualization, Writing – review & editing. FA: Data curation, Formal Analysis, Investigation, Methodology, Visualization, Writing – review & editing. KWi: Formal Analysis, Investigation, Writing – review & editing. TS: Data curation, Formal Analysis, Investigation, Methodology, Visualization, Writing – review & editing. NB: Investigation, Methodology, Validation, Writing – review & editing. GG: Writing – review & editing. KWe: Formal Analysis, Investigation, Methodology, Validation, Writing – review & editing. DH: Formal Analysis, Investigation, Methodology, Validation, Writing – review & editing. MB: Writing – review & editing. WB: Conceptualization, Funding acquisition, Writing – review & editing. MK-B: Conceptualization, Formal Analysis, Funding acquisition, Investigation, Methodology, Project administration, Resources, Supervision, Validation, Visualization, Writing – original draft, Writing – review & editing. CS: Conceptualization, Data curation, Formal Analysis, Investigation, Methodology, Project administration, Supervision, Visualization, Writing – original draft, Writing – review & editing.
