# Supplementary material for: Characterization of young and aged ferrets as animal models for SARS-CoV-2 infection with focus on neutrophil extracellular traps
Source: Front Immunol. 2023 Dec 15;14:1283595. doi: 10.3389/fimmu.2023.1283595 (PMC10758425; doi:10.3389/fimmu.2023.1283595)
Supplement: Supplementary file 1 [file DataSheet_1.pdf]

frontiers

Supplementary Material

https://www.frontiersin.org/articles/10.3389/fimmu.2023.1283595/abstract

doi: 10.3389/fimmu.2023.1283595

Characterization of young and aged ferrets as animal models for SARS-CoV-2 infection with focus on neutrophil extracellular traps

Veronika Pilchová[1,2], Ingo Gerhauser[3,4], Federico Armando[3], Katrin Wirz[1,2], Tom Schreiner[3,4], Nicole de Buhr[1,2], Gülşah Gabriel[5,6], Kerstin Wernike[7], Donata Hoffmann[7], Martin Beer[7], Wolfgang Baumgärtner[3,4], Maren von Köckritz-Blickwede[1,2*] and Claudia Schulz[1*]

[1]Research Center for Emerging Infections and Zoonoses (RIZ), University of Veterinary Medicine Hannover, Foundation, Hannover, Germany

[2]Institute of Biochemistry, University of Veterinary Medicine Hannover, Foundation, Hannover, Germany

[3]Department of Pathology, University of Veterinary Medicine Hannover, Foundation, Hannover, Germany

[4]Center for Systems Neuroscience Hannover (ZSN), University of Veterinary Medicine Hannover, Foundation, Hannover, Germany

[5]Department for Viral Zoonoses-One Health, Leibniz Institute of Virology, Hamburg, Germany

[6]Institute for Virology, University for Veterinary Medicine Hannover, Foundation, Hannover, Germany

[7]Institute of Diagnostic Virology, Friedrich Loeffler Institute, Greifswald-Insel Riems, Germany

*** Correspondence:**
Dr. med. vet. Claudia Schulz
claudia.schulz@tiho-hannover.de

Prof. Dr. rer. nat. Maren von Köckritz-Blickwede
maren.von.koeckritz-blickwede@tiho-hannover.de

1 Supplementary Tables and Figures

1.1 Supplementary Tables

Supplementary Table S1. An overview of animal age group, identification numbers and day of euthanasia in each experiment.

Experiment	Age	Ferret No.	Day of euthanasia
E#1	young	F1	control
	young	F2	control
	young	F3	control
	young	F4	4 dpi
	young	F5	4 dpi
	young	F6	4 dpi
	young	F7	7 dpi
	young	F8	7 dpi
	young	F9	7 dpi
	young	F10	21 dpi
	young	F11	21 dpi
	young	F12	21 dpi
E#2	aged	F13	control
	aged	F14	control
	aged	F15	control
	aged	F16	4 dpi
	aged	F17	4 dpi
	aged	F18	4 dpi
	aged	F19	7 dpi
	aged	F20	7 dpi
	aged	F21	7 dpi
	aged	F22	21 dpi
	aged	F23	21 dpi
	aged	F24	21 dpi

E#: Experiment, dpi: days post infection, control: uninfected control animals euthanized at -2 dpi

Supplementary Table S2. Results of health monitoring in pooled ferrets samples before the SARS-CoV-2 infection experiment.

Micro-organism	Pathogen	Matrix	Test	Result young	Result aged	Institution
Virus	*Ferret enteric coronavirus*	Feces	PCR	+	-	IDEXX
	Aleutian disease virus	Feces	PCR	-	-	IDEXX
	Rotavirus	Feces	Antigen IMC	-	nd	IDEXX
	Influenza A virus	Serum	competition ELISA (antibodies)	-	-	RIZ
	SARS-CoV-2	Nasal swab	PCR	-	-	RIZ
	SARS-CoV-2	Serum	indirect ELISA; NT (antibodies)	-	-	FLI
Bacteria	*Campylobacter coli*	Feces	PCR	-	-	IDEXX
	Campylobacter jejuni	Feces	PCR	-	-	IDEXX
	Lawsoni intracellularis	Feces	PCR	-	-	IDEXX
	Salmonella spp.	Feces	PCR	-	-	IDEXX
	Yersinia enterocolitica	Feces	PCR	-	-	IDEXX
	Bordetella bronchiseptica	Oral swab	PCR	-	-	IDEXX
	Pasteurella multocida	Oral swab	PCR	-	-	IDEXX
	Staphylococcus aureus	Oral swab	PCR	-	-	IDEXX
	Streptococcus pneumoniae	Oral swab	PCR	-	-	IDEXX
Parasite	*Cryptosporidium* spp.	Feces	Antigen ELISA	+	-	IDEXX
	Coccidia	Feces	Flotation	+#	-	IDEXX
	Giardia spp.	Feces	Antigen ELISA	-	-	IDEXX
	Otodectes spp.	Ear swab	microscopy	-	-	RIZ

IMC, immunochromatography; #, *Eimeria furonis* oocysts; nd, not done; IDEXX, veterinary diagnostic laboratory, IDEXX GmbH, Ludwigsburg, Germany; RIZ, Research Center for Emerging Infections and Zoonoses, University of Veterinary Medicine Hannover, Germany; FLI, Friedrich-Loeffler-Institut, Greifswald-Insel Riems, Germany.

Supplementary Table S3. Inflammatory lesions in the respiratory tract of aged ferrets.

Ferret ID	Group	Nose	Larynx	Trachea	Bronchi/ Bronchioli	Alveoli	Lung vessels
F13	Control	-	+	+	-	+	-
F14	Control	-	+	+	-	-	-
F15	Control	+	++	+	+	-	-
F16	4 dpi	++	+	+	+	+	+/++
F17	4 dpi	+	+	+	+	+	-
F18	4 dpi	+	+	+	+	-	+
F19	7 dpi	++	+/++	+	+	-	+
F20	7 dpi	++	+	+	+	++	+/++
F21	7 dpi	++	+	+	+	-	+
F22	21 dpi	+	-	+	+	+	+
F23	21 dpi	+	-	+	+	+	+
F24	21 dpi	++	+	+	+	+	+

- no inflammation; + mild inflammation; ++ moderate inflammation; dpi: days post infection.

Supplementary Table S4. Inflammatory lesions in extrarespiratory organs.

Ferret ID	Group	Organs
F13	Control	stomach +; intestine ++; liver ++; kidney +
F14	Control	conjunctiva ++; tear gland +; stomach +; intestine +/++; liver ++; thyroid gland ++; adrenal gland +++ **FRSCV**
F15	Control	conjunctiva +/++; stomach +/++; intestine +/++; liver +; kidney +; adrenal gland +
F16	4 dpi	conjunctiva ++; stomach ++; intestine +/++; liver ++/+++; kidney +; mesenterial lymph node +++ **FRSCV**
F17	4 dpi	conjunctiva +; stomach +; pancreas ++; intestine +/++; liver ++/+++; kidney +/++; meninx +
F18	4 dpi	conjunctiva +; stomach +/++; intestine +/++; liver +; kidney +; adrenal gland +
F19	7 dpi	myocardium +; tongue +; stomach +; pancreas +/++; intestine +/++; liver ++; kidney +
F20	7 dpi	conjunctiva +/++; mamma +; stomach +; intestine +/++; liver ++/+++; kidney +; thyroid gland +
F21	7 dpi	conjunctiva ++; tear gland +/++; stomach ++; intestine +/++; liver +; kidney +
F22	21 dpi	conjunctiva +/++; pancreas +/++; intestine +/++; liver +++; kidney +
F23	21 dpi	conjunctiva +; stomach +; intestine +/++; liver ++; kidney +
F24	21 dpi	conjunctiva +; stomach +; pancreas +; intestine +/++; liver +; kidney +

+ mild inflammation; ++ moderate inflammation; +++ severe inflammation; dpi: days post infection; FRSCV: ferret systemic coronavirus antigen detection by immunohistochemistry (see F14 in Suppl. Figure S8).

1.2 Supplementary Figures

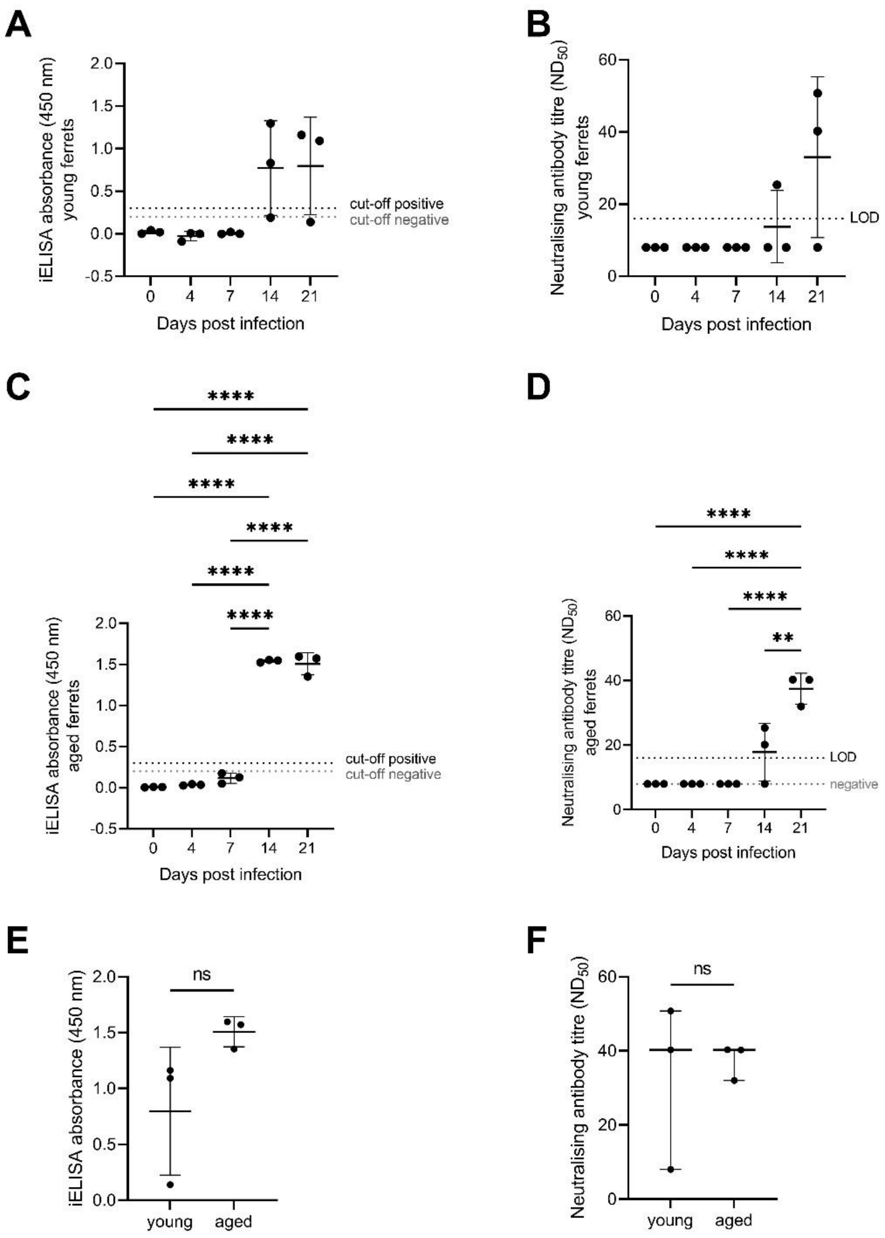

Supplementary Figure S1. Serological results. SARS-CoV-2 specific antibodies in ferrets before infection and at 4, 7, (A-D) and 21 (A-F) days post infection (dpi) after intratracheal SARS-CoV-2 infection determined with multispecies indirect ELISA (iELISA) (A, C, E) and neutralization test (NT) (B, D, F). Statistically analysis was performed with One-way ANOVA (A-D) or unpaired t-test (E, F). Whiskers represent mean and standard deviation. ND_{50}, neutralizing antibody titer 50%. *: $p \leq 0.05$. **: $p \leq 0.01$. ***: $p \leq 0.001$. ****: $p \leq 0.0001$. Ns: not significant.

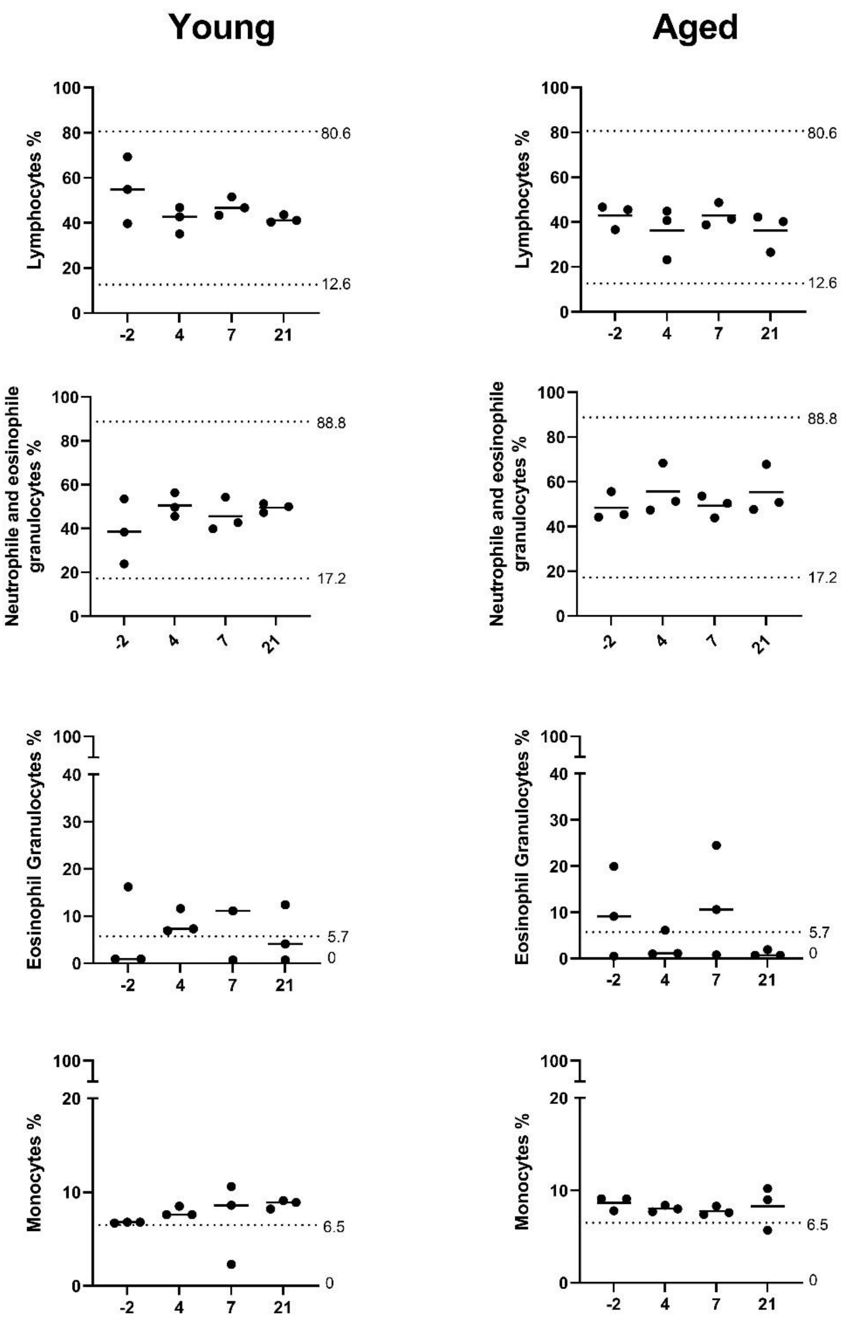

Supplementary Figure S2A. Hematological results. Differential blood cell count (%) of lymphocytes, granulocytes and monocytes automatically analyzed in EDTA whole-blood from young and aged ferrets before and after intratracheal infection with 10^6 $TCID_{50}$/mL SARS-CoV-2. Dotted lines show reference values for healthy ferrets that were obtained from Fehr et al. 2014. Crossbars represent the mean.

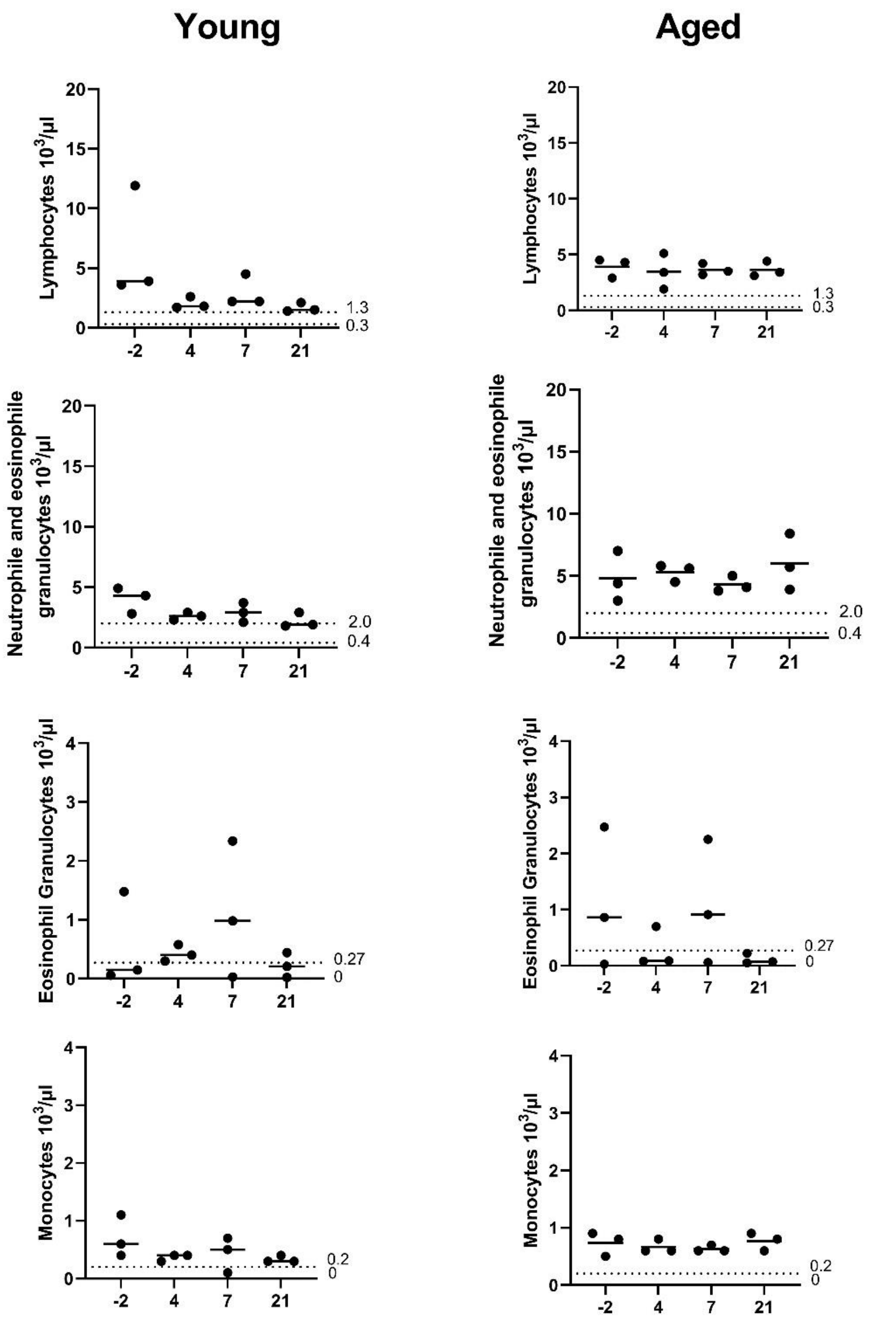

Supplementary Figure S2B. Hematological results. Number of lymphocytes, granulocytes and monocytes per µL automatically analyzed in EDTA whole-blood from young and aged ferrets before and after intratracheal infection with 10^6 $TCID_{50}$/mL SARS-CoV-2. Dotted lines show reference values for healthy ferrets that were provided by the manufacturer (Scilvet) of the hematology device (Vet abc plus). Crossbars represent the mean.

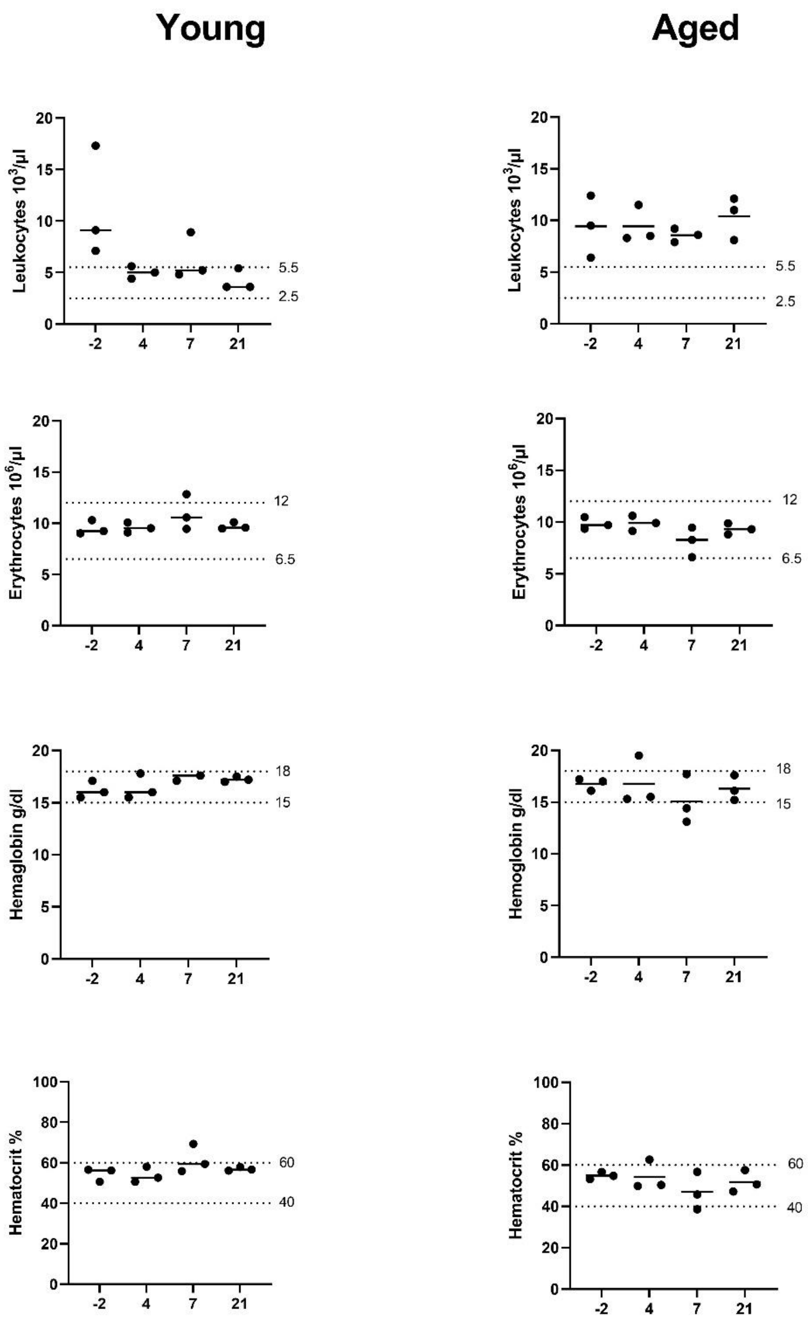

Supplementary Figure S2C. Hematological results. Number of leukocytes and erythrocytes as well as hemoglobin content and hematocrit (%) automatically analyzed in EDTA whole-blood from young and aged ferrets before and after intratracheal infection with 10^6 $TCID_{50}$/mL SARS-CoV-2. Dotted lines show reference values for healthy ferrets that were provided by the manufacturer (Scilvet) of the hematology device (Vet abc plus). Crossbars represent the mean.

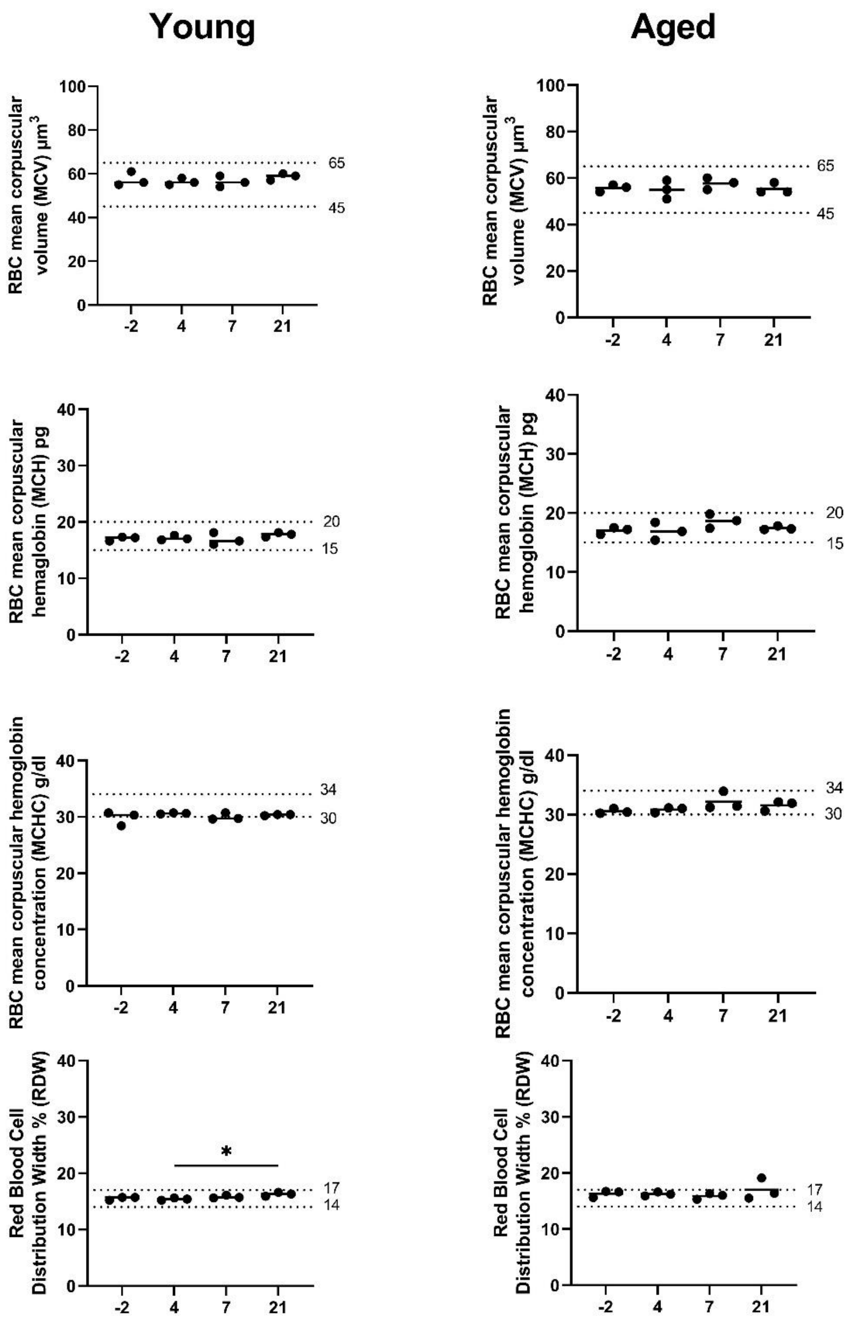

Supplementary Figure S2D. Hematological results. Erythrocyte (red blood cell, RBC) mean corpuscular hemoglobin and hemoglobin concentration and distribution width automatically analyzed in EDTA whole-blood from young and aged ferrets before and after intratracheal infection with 10^6 $TCID_{50}$/mL SARS-CoV-2. Dotted lines show reference values for healthy ferrets that were provided by the manufacturer (Scilvet) of the hematology device (Vet abc plus). Crossbars represent the mean. *: $p \leq 0.05$.

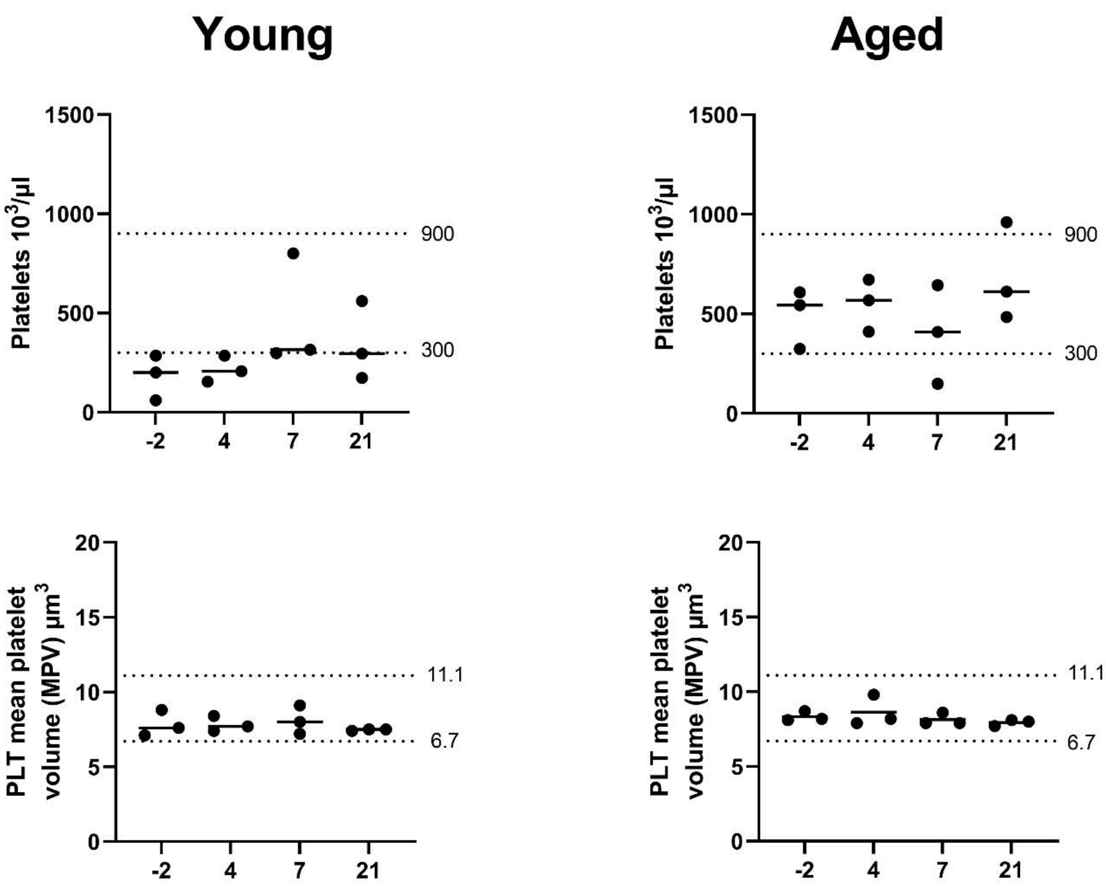

Supplementary Figure S2E. Hematological results. Number and mean volume of platelets automatically analyzed in EDTA whole-blood from young and aged ferrets before and after intratracheal infection with 10^6 $TCID_{50}$/mL SARS-CoV-2. Dotted lines show reference values for healthy ferrets that were provided by the manufacturer (Scilvet) of the hematology device (Vet abc plus). Crossbars represent the mean.

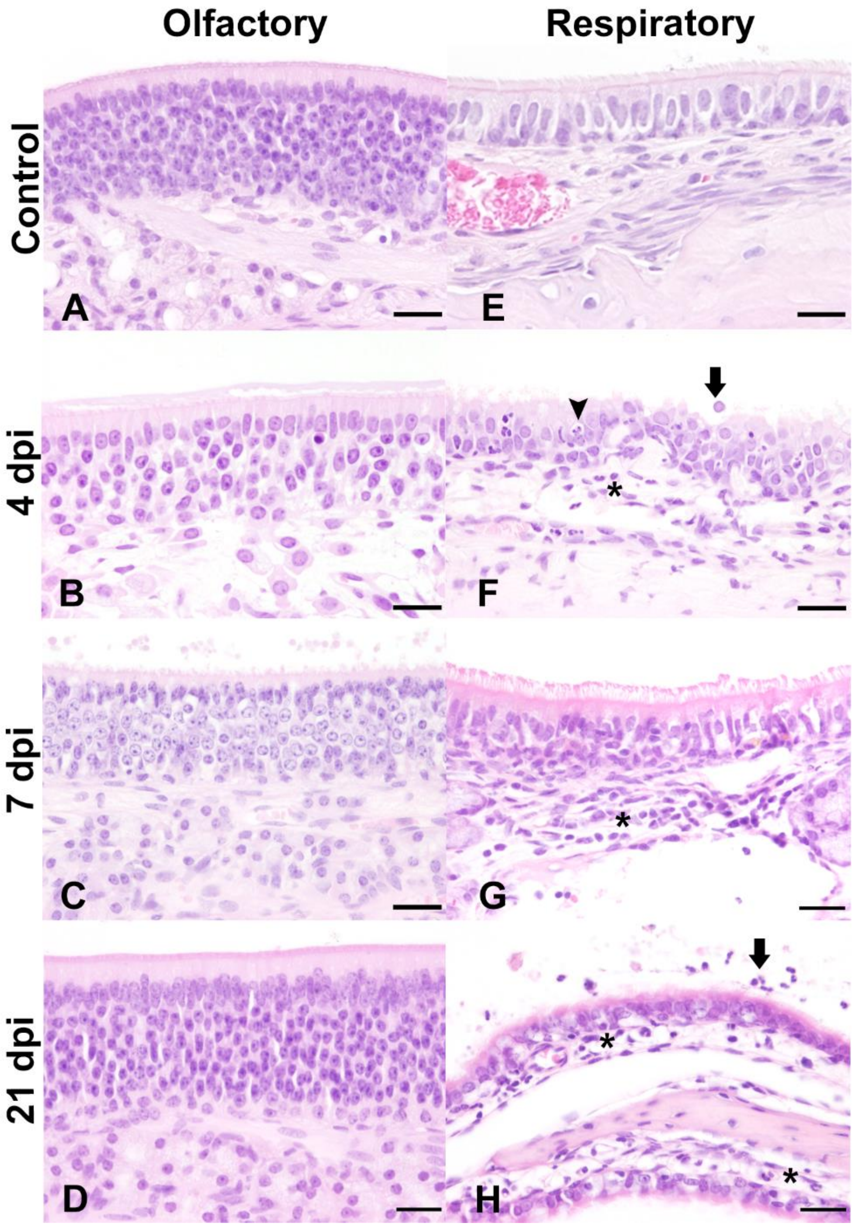

Supplemental Figure S3. Inflammatory lesions in the nasal cavity are restricted to the rostral respiratory epithelium in SARS-CoV-2-infected aged ferrets. Hematoxylin and eosin (HE) staining. **A-D.** Olfactory epithelium was intact at all investigated time points in SARS-CoV-2-infected ferrets. **A.** F14, control; **B.** F17, 4 days post infection (dpi); **C.** F20, 7 dpi; **D.** F22, 21 dpi. **E-H.** Respiratory epithelium is infiltrated by a moderate number of lymphocytes, macrophages, plasma cells and few neutrophils with single cell death and intraluminal cell debris. **E.** Intact multiciliated respiratory epithelium in a control ferret (F13). **F.** Respiratory epithelium in a SARS-CoV-2-infected ferret at 4 dpi (F17) (arrowhead: single cell death; asterisk: subepithelial inflammatory cells; arrow: epithelial necrosis with ciliary loss and intraluminal cellular debris). **G.** Respiratory epithelium in a SARS-CoV-2-infected ferret at 7 dpi (F20) (asterisk: subepithelial lymphocytes, macrophages, plasma cells and few neutrophils). **H.** Respiratory epithelium in a SARS-CoV-2-infected ferret at 21 dpi (F23) (asterisk: subepithelial lymphocytes, macrophages, plasma cells and few neutrophils; arrow: intraluminal inflammatory cells and cellular debris). Bars: 20 μm.

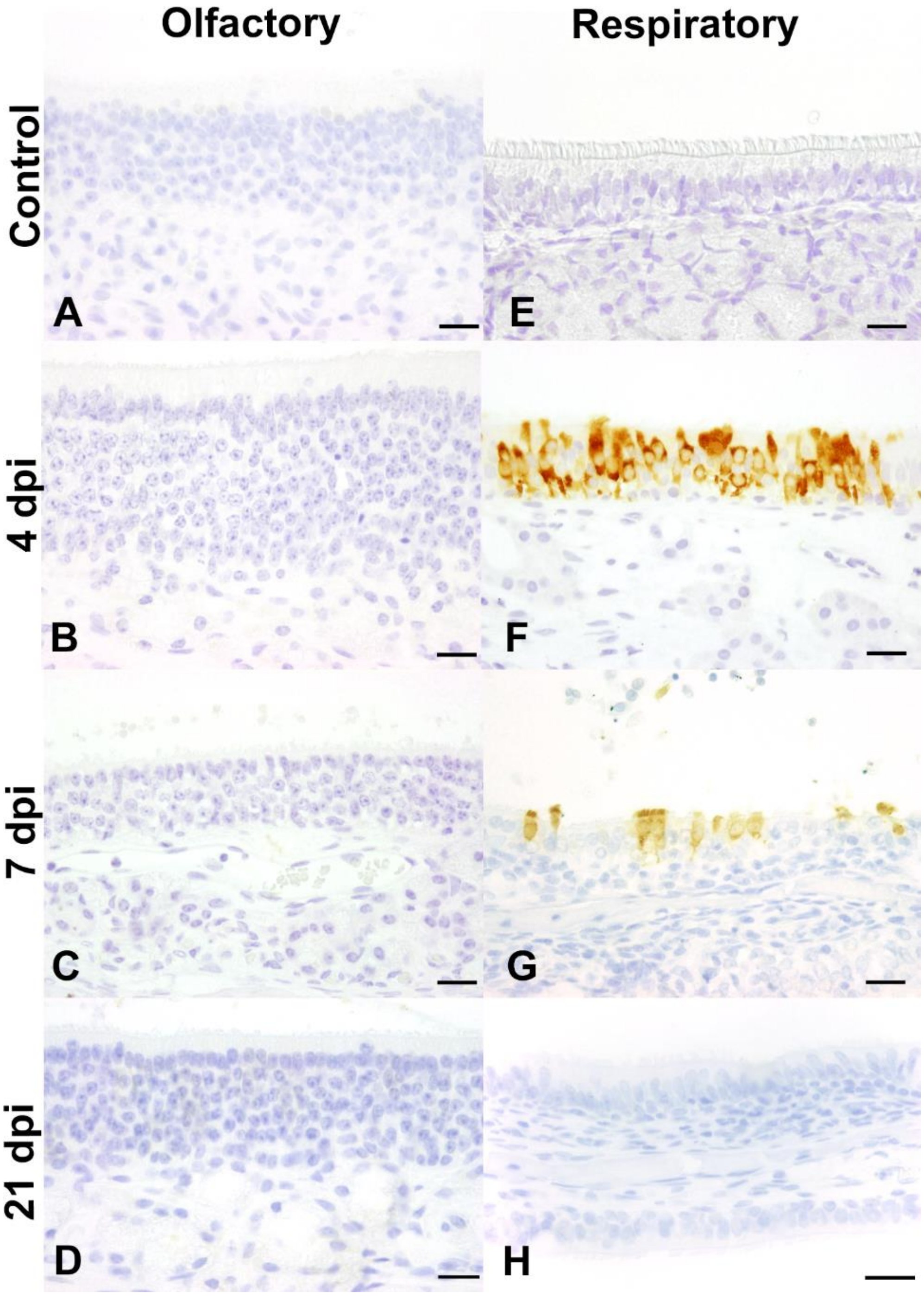

Supplementary Figure S4. Immunohistochemistry of the nasal cavity of SARS-CoV-2-infected aged ferrets. **A**-**D.** Olfactory epithelium. No viral antigen was detected in the olfactory epithelium at all investigated time points. **A.** F13, control; **B.** F16, 4 days post infection (dpi); **C:** F20, 7 dpi; **D.** F22, 21 dpi. **E-H.** Respiratory epithelium. SARS-CoV-2-nucleoprotein (NP) was detected in multiciliated cells in the nasal cavity of all SARS-CoV-2-infected ferrets at 4 dpi (**F**, F17) and 7 dpi (**G**, F19). No viral antigen was detected in control animals (**A**, F13) or animals euthanized at 21 dpi (**H**, F22). SARS-CoV-2 NP immunohistochemistry using 3,3'-diaminobenzidine (DAB) as chromogen and Mayer's hematoxylin as counterstaining. Bars: 20 μm.

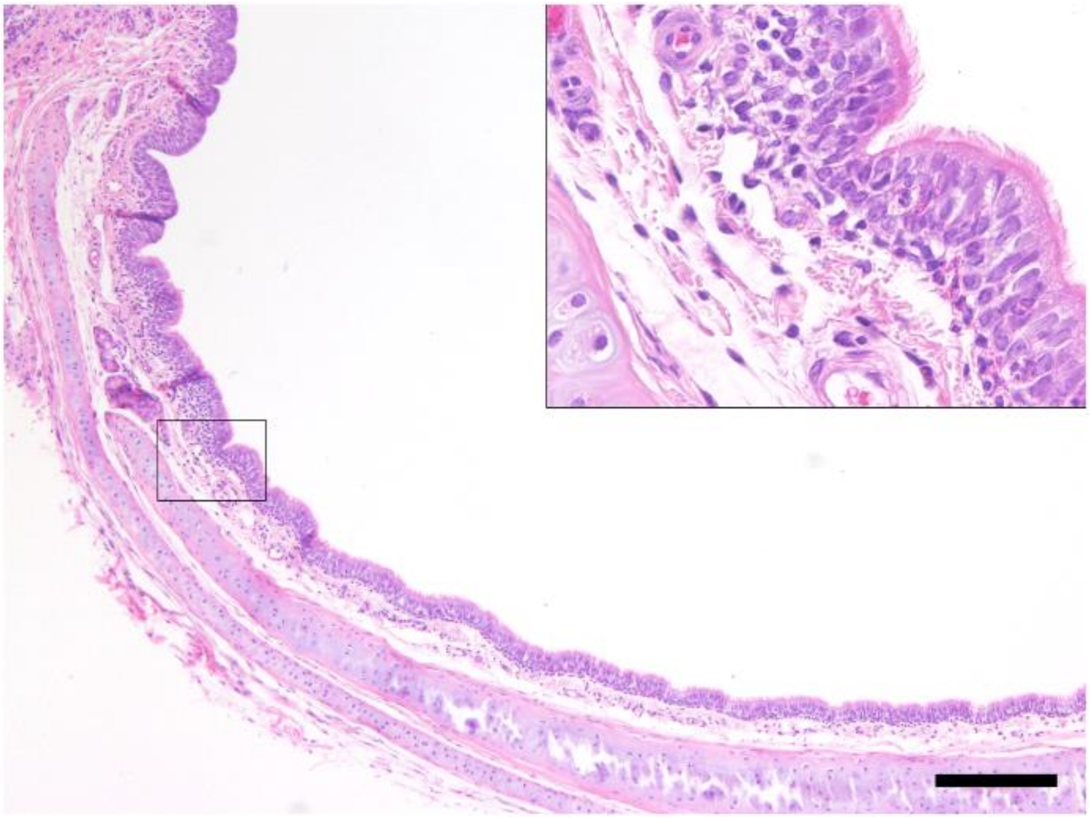

Supplementary Figure S5. Tracheitis, trachea, SARS-CoV-2 infected ferret at 4 days post infection (F17). Hematoxylin and eosin (HE) staining. The mucosa is infiltrate by few lymphocyte, macrophages and eosinophils. Inset: higher magnification. Bar = 200 µm (inset: bar = 50 µm).

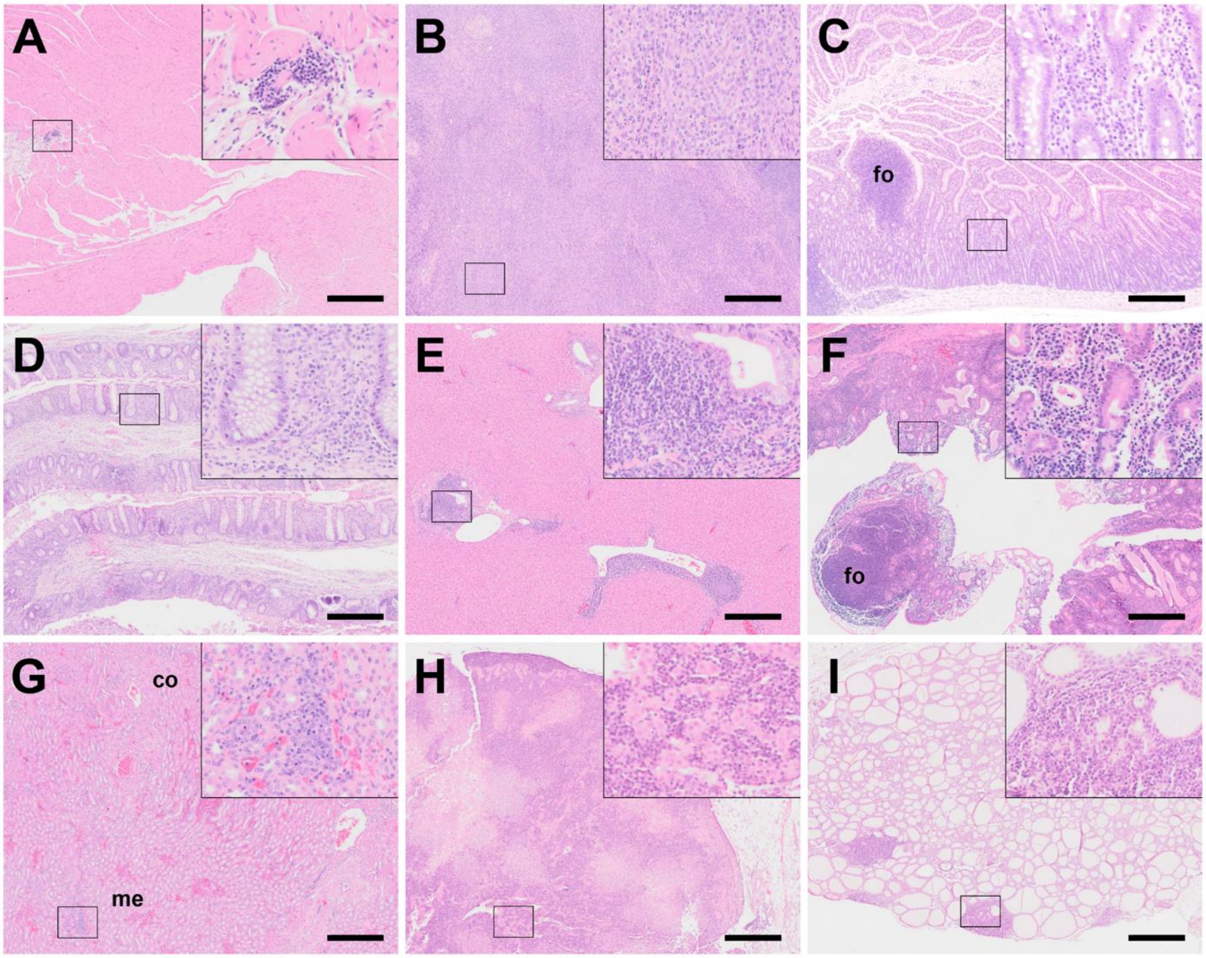

Supplementary Figure S6. Inflammatory lesions in the heart, mesenterial lymph node, gastrointestinal tract, pancreas, liver, gall bladder, kidney, adrenal gland, and thyroid gland of control and SARS-CoV-2 infected ferrets. Hematoxylin and eosin (HE) staining. **A.** Myocarditis, SARS-CoV-2 infected ferret at 7 days post infection (dpi, F19). There is a focal infiltration of the myocardium with several lymphocytes and few macrophages. Inset: higher magnification. **B.** Lymphadenitis, mesenterial lymph node, SARS-CoV-2 infected ferret at 4 dpi (F16). The tissue is replaced in a large area by many macrophages and neutrophils forming granulomas. Immunohistochemistry showed intralesional coronavirus antigen characteristic for ferret systemic coronavirus (FRSCV)-associated disease (see Suppl. Figure S5). Inset: higher magnification of pyogranulomatous inflammation. **C.** Enteritis, jejunum, SARS-CoV-2 infected ferret at 21 dpi (F23). The mucosa is infiltrated by many plasma cells and few lymphocytes and macrophages. Note large follicular structure (fo) of the mucosa-associated lymphoid tissue (MALT). Inset: Higher magnification of inflammatory cells in the mucosa. **D.** Colitis, colon, SARS-CoV-2 infected ferret at 7 dpi (F20). The mucosa is diffusely infiltrated by lymphocytes and macrophages and few plasma cells and neutrophils. Inset: Higher magnification of inflammatory cells in the mucosa. **E.** Hepatitis, control ferret (F13). The portal areas are infiltrated by many lymphocytes and several macrophages partly forming follicular structures. Inset: Higher magnification of inflammatory cells in the portal area. **F.** Cholecystitis, gall bladder, SARS-CoV-2 infected ferret at 4 dpi (F17). The mucosa is severely hyperplastic and infiltrated by abundant lymphocytes and plasma

cells partly forming follicular structures (fo). Inset: Higher magnification of inflammatory cells in the mucosa. **G.** Nephritis, kidney, SARS-CoV-2 infected ferret at 21 dpi (F23). There is a focal infiltration of the interstitium by several lymphocytes and macrophages in the renal medulla (me). Lack of inflammatory cells in the renal cortex (co). Inset: Higher magnification of inflammatory cells between tubular structures. **H.** Adrenalitis, adrenal gland, control ferret (F14). The tissue is infiltrated by many lymphocytes and macrophages as well as few neutrophils and plasma cells destroying the original tissue that also contains large areas of necrosis. Immunohistochemistry demonstrated intralesional coronavirus antigen characteristic for FRSCV-associated disease (see Suppl. Figure S8). Inset: higher magnification of inflammatory cells. **I.** Thyroiditis, thyroid gland, control ferret (F14). There is a multifocal infiltration of the parenchyma by many lymphocytes and macrophages forming follicular-like structures. Inset: higher magnification of inflammatory cells between thyroid follicles. Bars = 400 µm (insets: bars = 100 µm).

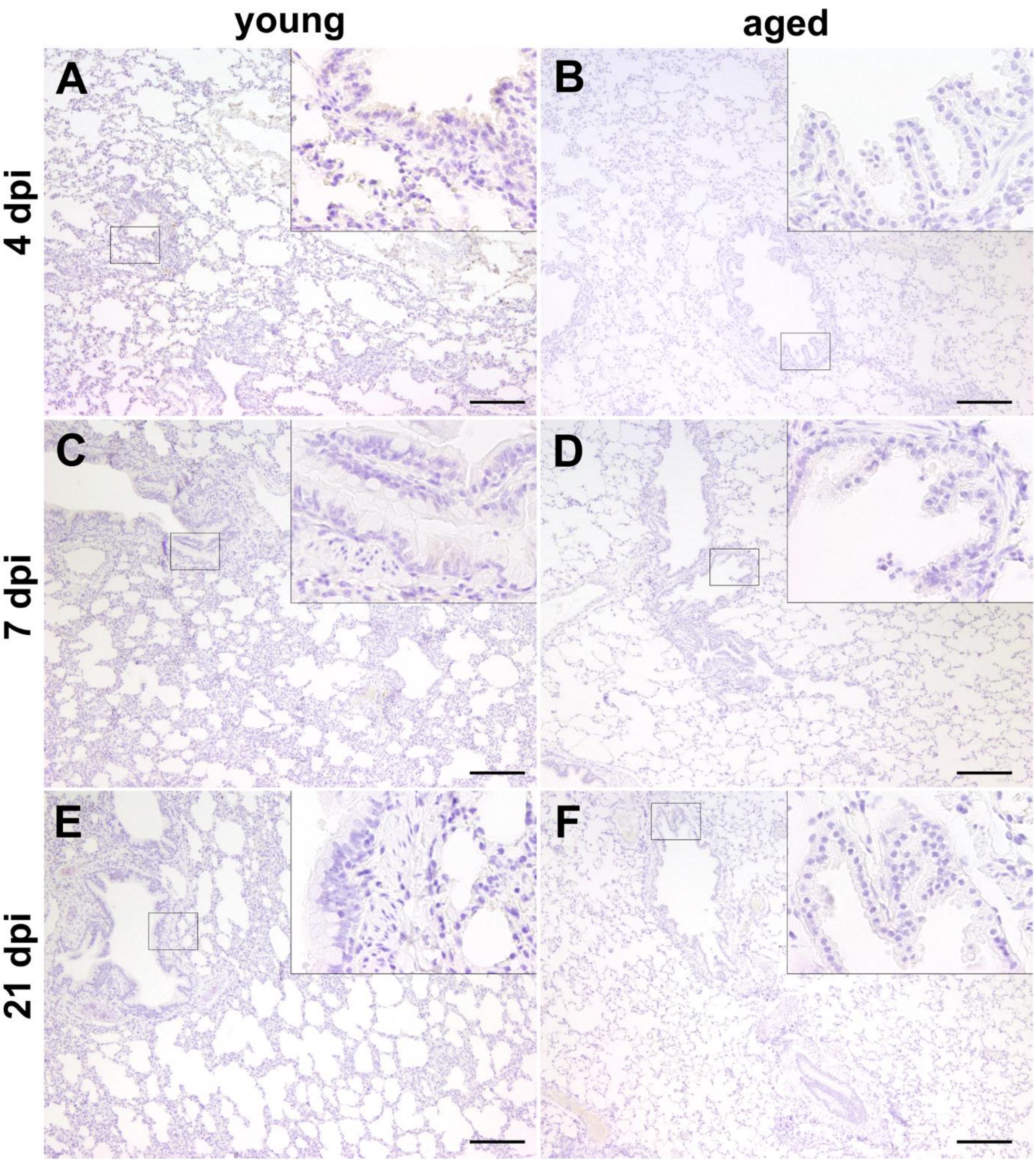

Supplementary Figure S7. Severe acute respiratory syndrome coronavirus (SARS-CoV)-2 infection, lung, ferret, 4, 7, and 21 days post infection (dpi). Immunohistochemistry. No positive signal for SARS-CoV-2 nucleoprotein detected in the lung (young: **A**, F6; **C**, F8; **E**, F10; aged: **B**, F17; **D**, F19; **F**, F22). Higher magnifications show details of the areas enclosed by the rectangles including bronchiolar epithelial cells. Bars = 200 µm (insets: bars = 50 µm).

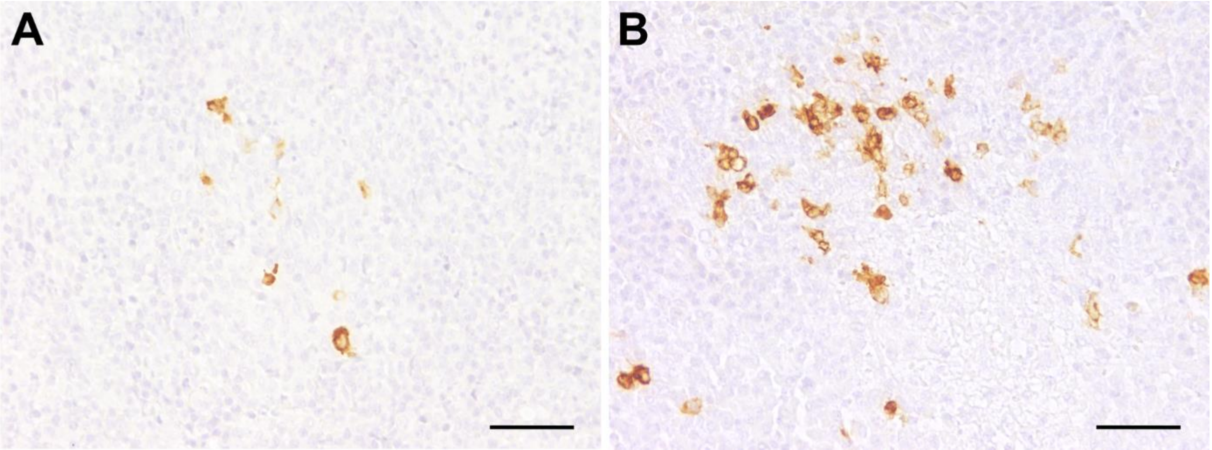

Supplementary Figure S8. Ferret systemic coronavirus (FRSCV)-associated disease, ferret. Immunohistochemistry. **A.** Mesenterial lymph node. Coronavirus antigen (brown signal) detected in macrophages of a SARS-CoV-2 infected ferret at 4 dpi (F16; same animal as shown in Suppl. Figure S6B). **B.** Adrenal gland. Coronavirus antigen (brown signal) detected in macrophages of a control ferret (F14; same animal as shown in Suppl. Figure S6H). Bars = 50 µm.

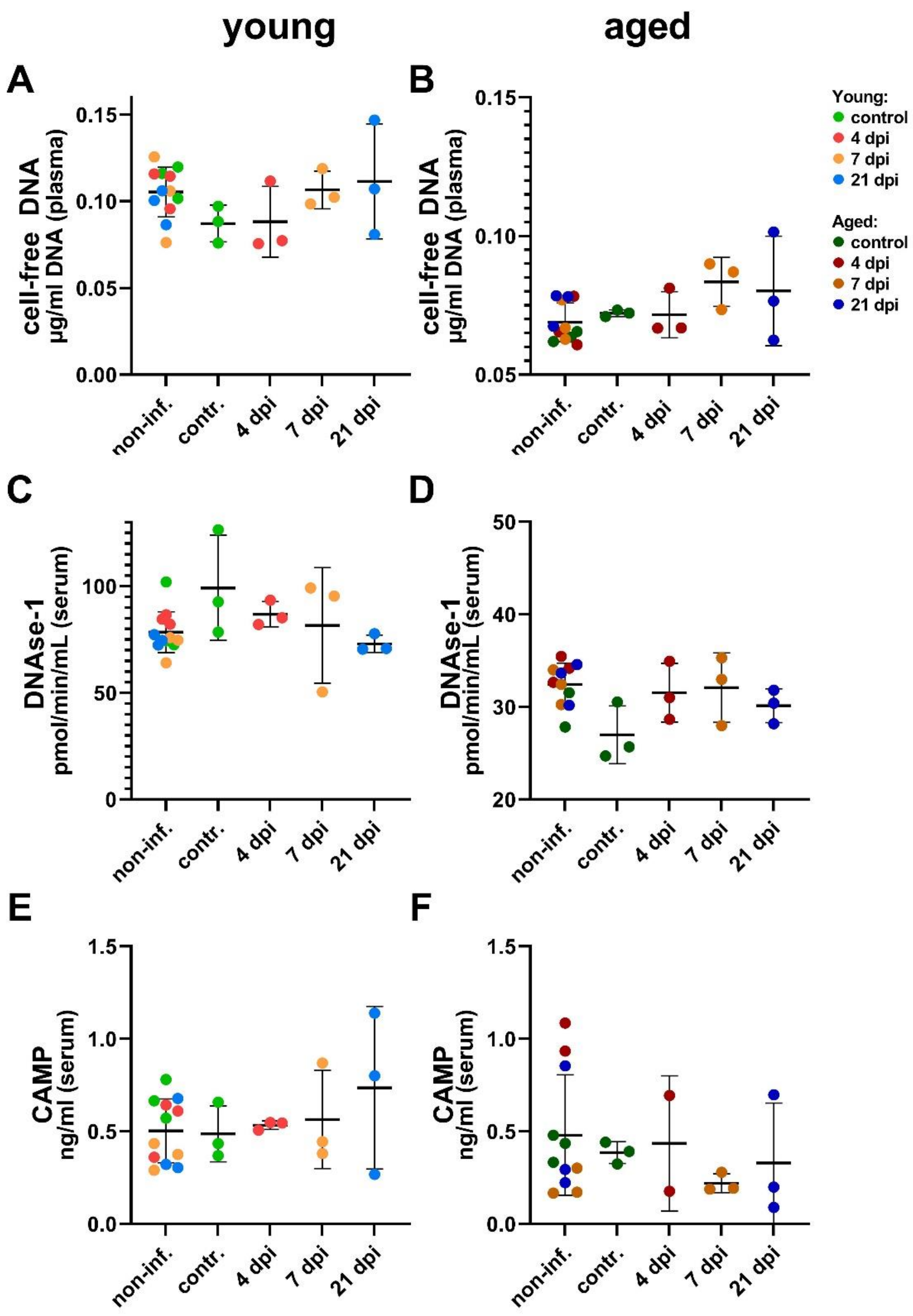

Supplementary Figure S9. Detection of NET markers in serum of young and aged ferrets. **A.** Cell-free DNA in young ferrets. **B.** Cell-free DNA in aged ferrets. C. DNase-1 activity in young. **D.** DNase-1 activity in aged. **E.** Cathelicidin antimicrobial peptide (CAMP) ELISA in young. **F.** CAMP ELISA in aged. Non-inf.: serum from all animals before infection. Contr.: serum from 3 control animals euthanized 2 days before infection. Data were analyzed with an unpaired two-tailed one-way ANOVA. Whiskers represent mean and standard deviation.

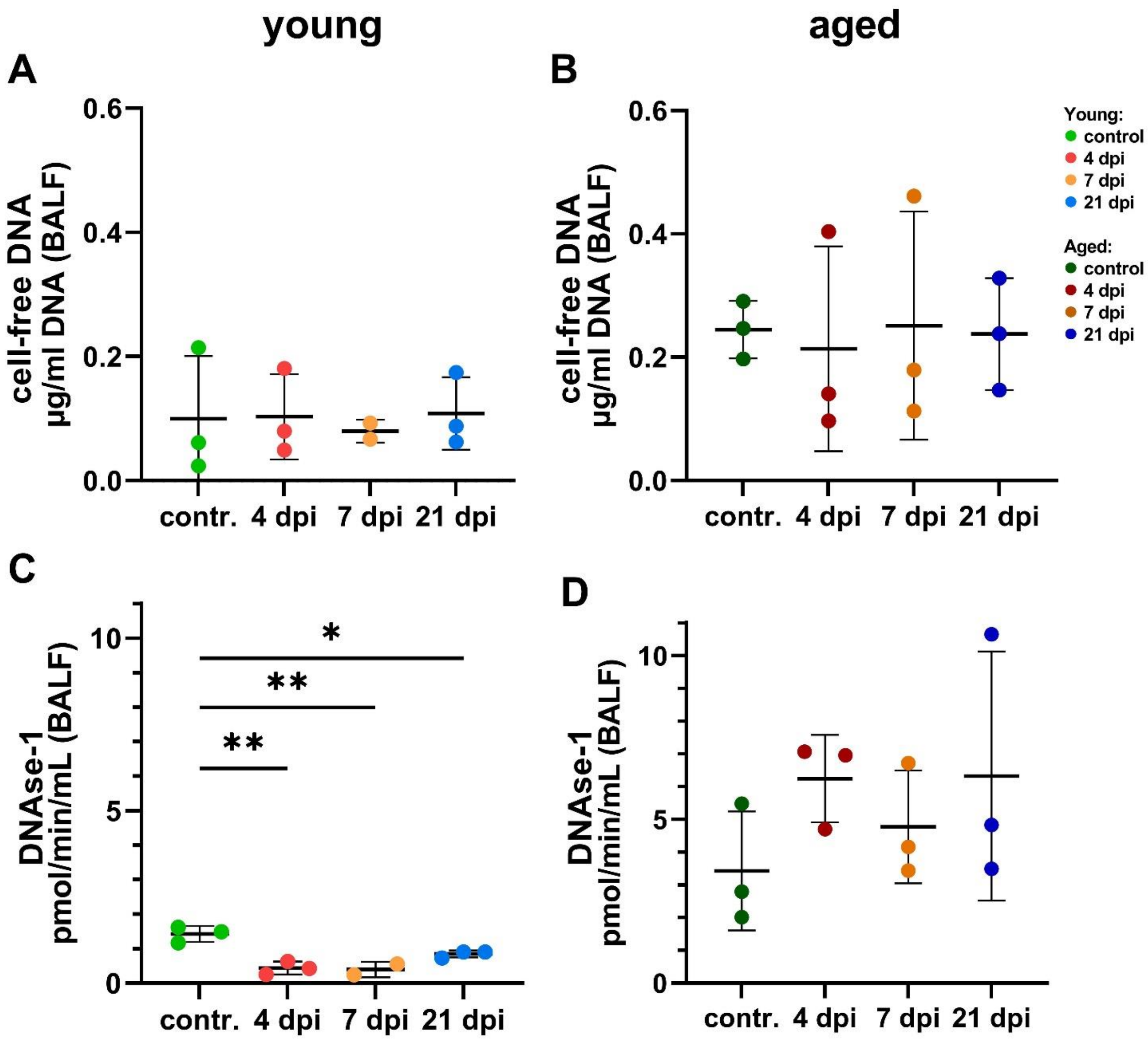

Supplementary Figure S10. Detection of NET markers in bronchioalveolar lavage fluid (BALF) of young and aged ferrets. **A.** Cell-free DNA in young ferrets. **B.** Cell-free DNA in aged ferrets. **C.** DNase-1 activity in young ferrets. **D.** DNase-1 activity in aged animals. Non-inf.: BALF from all animals before infection. Contr.: BALF from 3 control animals euthanized 2 days before infection. Data were analyzed with an unpaired two-tailed one-way ANOVA test. Whiskers represent mean and standard deviation. *: $p \leq 0.05$. **: $p \leq 0.01$.

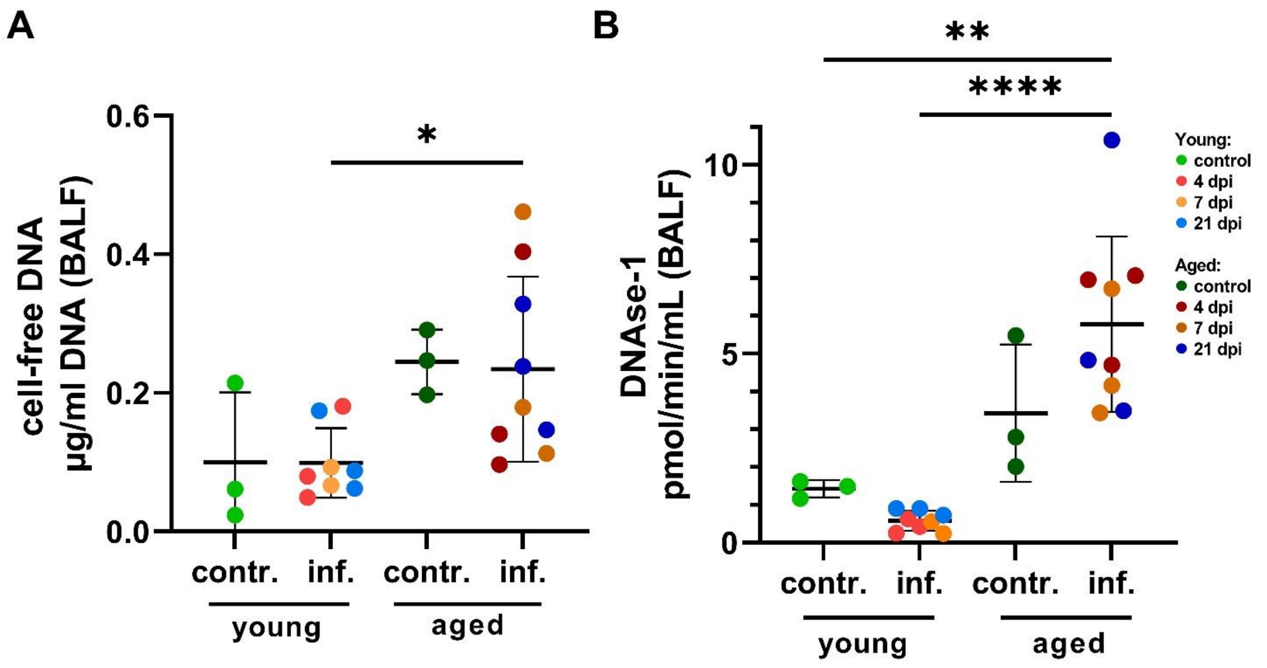

Supplementary Figure S11. Comparison of NET marker detection in bronchoalveolar lavage fluid (BALF) between young and aged ferrets. **A.** Cell-free DNA. **B.** DNase-1 activity. Data were analyzed with an unpaired two-tailed one-way ANOVA. Whiskers represent mean and standard deviation. *: $p \leq 0.05$. **: $p \leq 0.01$. ***: $p \leq 0.001$. ****: $p \leq 0.0001$.

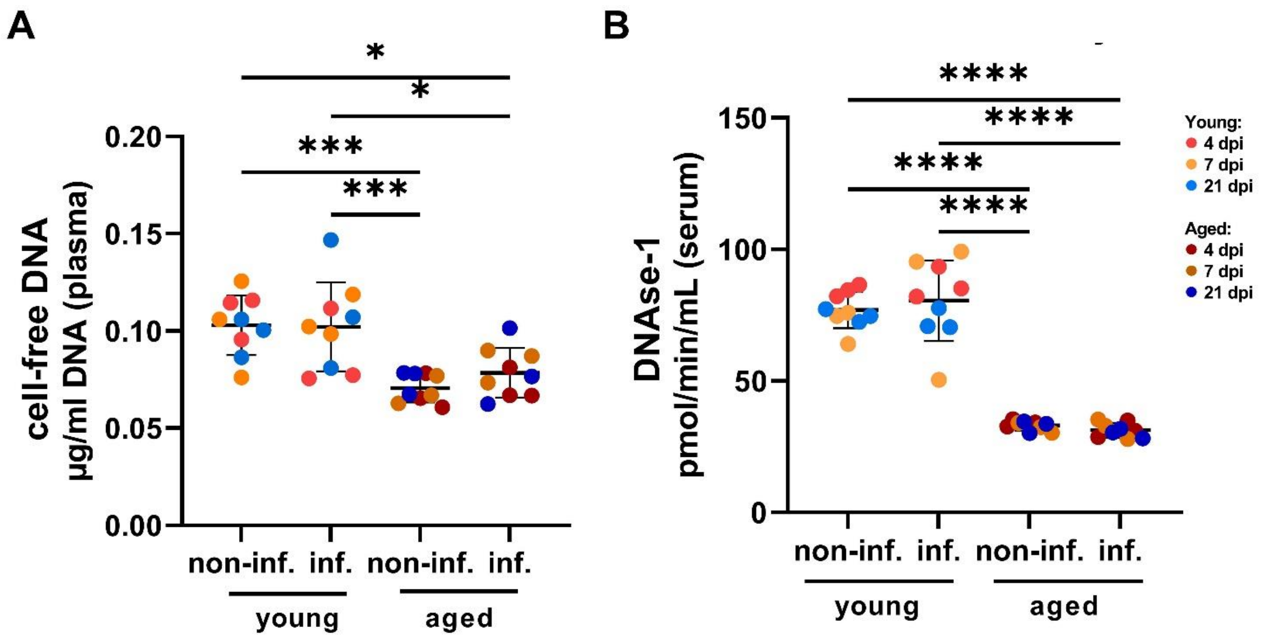

Supplementary Figure S12. Comparison of NET marker detection in plasma/serum between young and aged ferrets. **A.** Cell-free DNA. **B.** DNase-1 activity. Data were analyzed with an unpaired two-tailed one-way ANOVA. Whiskers represent mean and standard deviation. *: $p \leq 0.05$. **: $p \leq 0.01$. ***: $p \leq 0.001$. ****: $p \leq 0.0001$.

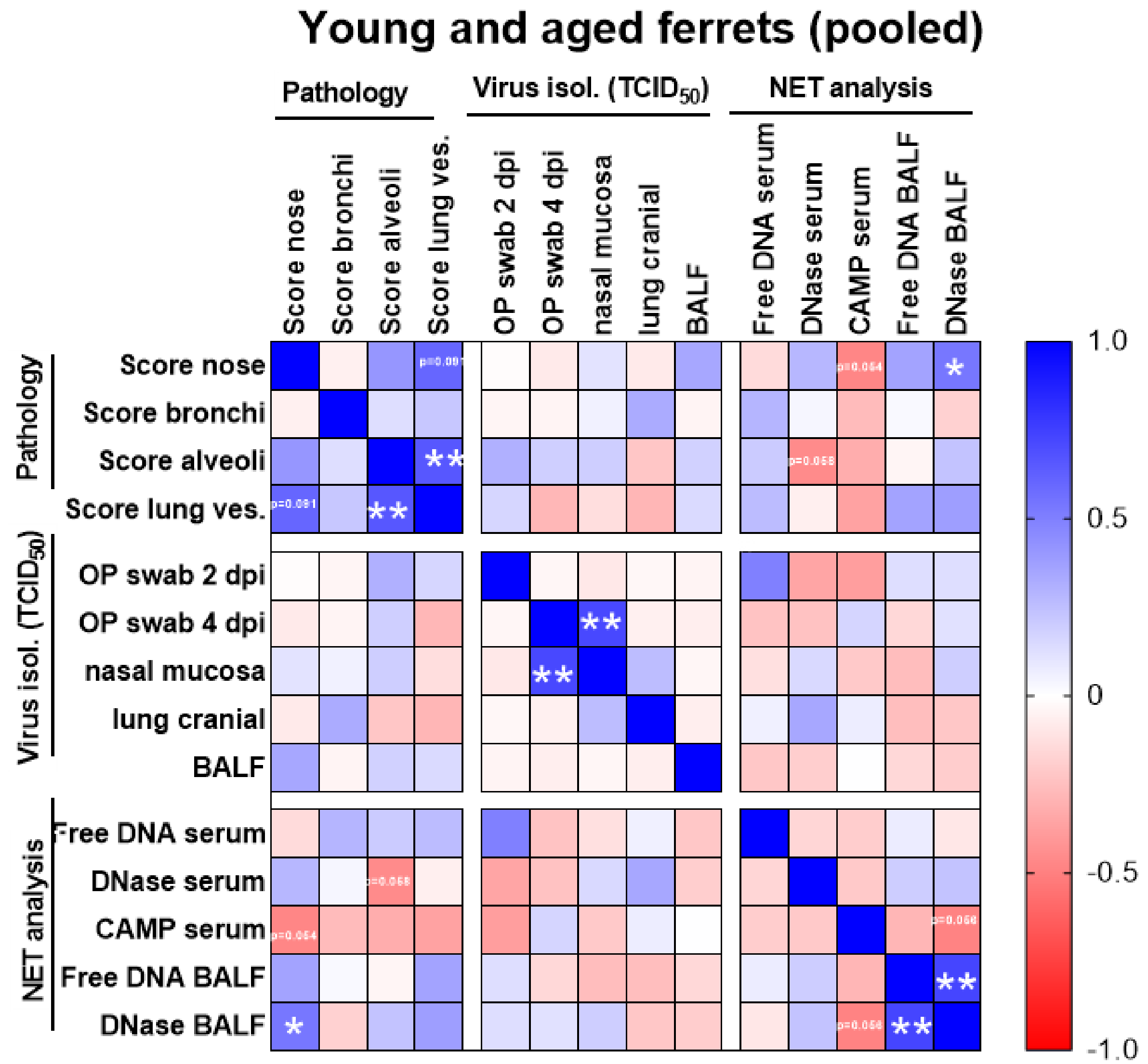

Supplementary Figure S13. Correlation matrix of histopathological scores, virus titers from end-point dilution assay and results from NET marker analysis. Histopathological scores were obtained from nose, lung bronchi, alveoli and vessel lesions, and virus titers from oropharyngeal (OP) swabs at 2 and 4 days post infection (dpi), nasal mucosa, lung cranial and bronchoalveolar lavage fluid (BALF). Cell-free DNA, DNase-1 activity and CAMP concentration were analysed in serum and cell-free DNA and DNase-1 activity in BALF. Data were analyzed with parametric Pearson´s two-tailed correlation with 95% confidence interval. Only data from young and aged (pooled) infected animals (4-21 dpi) were included in the analysis. Results with p values between 0.05 and 0.1 are directly shown in the figure. *: $p \leq 0.05$. **: $p \leq 0.01$.
